# NF-κB c-Rel is a critical regulator of TLR7-induced inflammation in psoriasis

**DOI:** 10.1016/j.ebiom.2024.105452

**Published:** 2024-11-24

**Authors:** Angela Rose Liu, Nandini Sarkar, Jordan D. Cress, Tristan J. de Jesus, Ananya Vadlakonda, Joshua T. Centore, Alexis D. Griffith, Bethany Rohr, Thomas S. McCormick, Kevin D. Cooper, Parameswaran Ramakrishnan

**Affiliations:** a Department of Pathology, Case Western Reserve University, 2103 Cornell Road, Cleveland, Ohio 44106, USA; b The Case Comprehensive Cancer Center, Case Western Reserve University, 2103 Cornell Road, Cleveland, Ohio 44106, USA; c Department of Biochemistry, Case Western Reserve University, 2109 Adelbert Road, Cleveland, Ohio 44106, USA; d Department of Dermatology, Case Western Reserve University, 2109 Adelbert Road, Cleveland, Ohio 44106, USA; e University Hospitals-Cleveland Medical Center, 11100 Euclid Ave, Cleveland, Ohio 44106, USA; f Louis Stokes Veterans Affairs Medical Center, 10701 East Blvd, Cleveland, Ohio 44106, USA

**Keywords:** TLR7, NF-κB c-Rel, Inflammation, Psoriasis, Transcription

## Abstract

**Background:**

Nuclear factor kappa B (NF-κB) c-Rel is a psoriasis susceptibility locus, however mechanisms underlying c-Rel transactivation during disease are poorly understood. Inflammation in psoriasis can be triggered following Toll-like Receptor 7 (TLR7) signalling in dendritic cells (DCs), and c-Rel is a critical regulator of DC function. Here, we studied the mechanism of TLR7-induced c-Rel-mediated inflammation in DCs.

**Methods:**

The overall expression of c-Rel was analysed in skin sections from patients with psoriasis in human transcriptomics datasets as well as the imiquimod-induced psoriasis mouse model. The function of c-Rel in DCs following TLR7 stimulation was determined by c-Rel CRISPR/Cas9 knockout DC2.4 immortalised cells and primary bone marrow derived dendritic cells from c-Rel knockout C57BL6/J mice.

**Findings:**

c-Rel is highly expressed in lesional skin of patients with psoriasis and TLR7-induced psoriatic lesions in mice. c-Rel deficiency protected mice from the disease, and specifically compromised TLR7-induced, and not TLR9- or TLR3-induced, inflammation in dendritic cells. Mechanistically, c-Rel deficiency disrupted activating NF-κB dimers and allowed binding of inhibitory NF-κB homodimers to the IL-1β and IL-6 promoters thus inhibiting their expression. This functionally compromises the ability of c-Rel deficient DCs to induce Th17 polarisation, which is critical in psoriasis pathogenesis.

**Interpretation:**

Our findings reveal that c-Rel is a key regulator of TLR7-mediated dendritic cell-dependent inflammation, and that targeting c-Rel-dependent signalling could prove an effective strategy to dampen excessive inflammation in TLR7-related skin inflammation.

**Funding:**

A complete list of funding sources that contributed to this study can be found in the Acknowledgements section.

**Research in context**

**Evidence before this study**

NF-κB c-Rel is a susceptibility locus for psoriasis and a critical regulator of dendritic cell (DC) function. DCs are key mediators in the development of psoriasis through the production of inflammatory cytokines and the activation of T cells. Recent papers have also reported that TLR7 signalling through the NF-κB pathway plays a key role in psoriasis disease progression.

**Added value of this study**

This work focuses on DCs, which are critical in psoriasis pathogenesis. We studied the TLR7/c-Rel signalling axis and delineated the molecular mechanism of TLR7-mediated DC functions. Using patient data, a psoriasis mouse model and comprehensive mechanistic studies, we showed that TLR7-induced c-Rel-dependent transcription in DCs regulates inflammatory cytokine production and Th17 cell differentiation that promote skin inflammation and psoriasis development.

**Implications of all the available evidence**

Understanding disease mechanism is important in identifying new targets for more effective disease treatment. The elucidation of the TLR7/c-Rel signalling axis in psoriasis pathogenesis warrants strategies that inhibit c-Rel activation to be a possible therapeutic approach for psoriasis treatment.

## Introduction

Psoriasis is a chronic inflammatory skin disease characterised by epidermal hyperplasia, excessive inflammatory cytokine production, and immune cell infiltration in the dermis.^1^ Dendritic cells (DCs) are key cell mediators in psoriasis pathogenesis through the production of inflammatory mediators and the induction of T cell activation and differentiation.^2^ The formation of a DC:T cell immunological synapse triggers keratinocyte proliferation and the formation of a psoriatic lesion.^3^ The importance of DCs in psoriasis development and maintenance has been widely emphasised, however the signalling mechanisms which regulate their involvement in psoriasis remain unclear.

DC function is regulated by multiple proteins, including by members of the nuclear factor kappa B (NF-κB) transcription factor family that act as central regulators of inflammation. The NF-κB family consists of the preformed proteins RelA (p65), RelB, c-Rel, NF-κB1 (p50/p105) and NF-κB2 (p52/100), all of which function as homo- or heterodimers. The canonical pathway consists of dimers containing NF-κB subunits, except the alternative pathway dimer RelB/p52. Dimers are sequestered in the cytoplasm and bound to the Inhibitor of kappa B (IκB) protein family members when cells are at rest. Stimulus-induced IκB degradation releases bound NF-κB dimers, allowing its translocation to the nucleus for gene transcription.^4^ NF-κB c-Rel is critical for cell type specific expression of several pro-inflammatory cytokines including IL-23p19^5^ and IL-12p35^6^ in DCs, IL-12p40 in macrophages^7^^,^^8^ and IL-2 in T cells.^9^^,^^10^ Previous studies have shown that c-Rel is vital for specific DC functions, i.e., allogeneic T cell activation,^11^ but not DC development^12^ or Toll-like receptor 4 (TLR4)-induced DC maturation.^11^ Deficiency of c-Rel in mice has been shown to be protective against rheumatoid arthritis^13^^,^^14^ and autoimmune encephalomyelitis,^15^ and partial knockdown of c-Rel using siRNA ameliorates imiquimod-induced psoriasiform hyperproliferation.^16^ On the other hand, c-Rel knockout mice show accelerated autoimmune diabetes pathogenesis^17^ and increased mortality due to polymicrobial sepsis associated with a lack of TLR4-induced c-Rel/p50 dimer-mediated IL-12 and IL-23 expression.^18^

Toll-like receptors (TLRs) are expressed on a variety of immune and nonimmune cells. TLRs 3, 7, 8 and 9 are mainly found within endosomes, where TLR3 recognises double-stranded RNA, TLR9 detects unmethylated DNA containing CpG motifs, and TLR7 and its structurally similar TLR8 recognise single-stranded RNA.^19^ TLR7 expression has been described in B cells and DCs.^20^, ^21^, ^22^ TLR7 is expressed basally on plasmacytoid dendritic cells,^20^^,^^21^ and cell stimulation upregulates the expression of TLR7 on myeloid dendritic cells^22^ and conventional dendritic cells^23^ with a corresponding secretion of proinflammatory cytokines. Imiquimod (IMQ), a synthetic TLR7 agonist, induces keratinocyte hyperplasia that resembles human plaque psoriasis in mouse models.^24^ Clinically, imiquimod is used as a topical treatment for genital warts^25^ and basal cell carcinoma.^26^ Usage of topical IMQ in patients can also inadvertently exacerbate previously controlled psoriasis in both IMQ-treated and uninvolved distant skin sites.^27^^,^^28^ TLR7, like other TLRs except TLR3, signals through the MyD88 adaptor protein, leading eventually to the activation of the NF-κB pathway, and resulting in the expression of inflammatory cytokines and chemokines. The importance of NF-κB signalling in TLR7-induced inflammation has been described,^29^ however the molecular mechanism involving specific NF-κB subunits is not well understood.

c-Rel has been shown to be a critical component of various TLR dependent pathways. c-Rel fails to be activated in MyD88 KO macrophages following TLR4 activation.^7^ The p65/p50 heterodimer is prevalent in immature DCs, while TLR4 activation primarily induces the c-Rel/p50 heterodimer in DCs,^12^ suggesting a ligand-dependent specificity of NF-κB subunit function. We previously showed that c-Rel-dependent inflammatory gene expression requires the adaptor protein Sam68 following TLR2 and TLR3 activation.^30^ The role of c-Rel has also been shown in TLR2, 3, 4, 6, and 9-induced activation of the IL-23p19 promoter,^5^ however there is a lack of knowledge regarding the potential role of c-Rel in TLR7 signalling, and the possibility of this signalling axis being involved in the development of psoriasis.

In this study, we studied the specific role of NF-κB c-Rel in TLR7 signalling in DCs and its functional relevance in psoriasis. Transcriptomics analysis of human skin biopsies identified NF-κB c-Rel expression to be elevated in the skin of patients with psoriasis, with expression levels substantially reduced following treatment. Consistent with these observations, genetic c-Rel deficiency reduced disease phenotype in a TLR7-induced psoriasis-like mouse model. We found that c-Rel function is critical in the transcriptional regulation of a subset of TLR7-induced inflammatory genes in DCs. Our mechanistic studies show that c-Rel, along with NF-κB p65, are key NF-κB subunits regulating TLR7 mediated inflammation. The absence of c-Rel results in the enrichment of the inhibitory p50 homodimer binding to the promoters of inflammatory genes, which likely contributes to the decreased production of inflammatory cytokines such as IL-1β and IL-6. Functionally, the decrease in DC cytokine production results in a decreased ability of c-Rel deficient cells to induce Th17 differentiation, which is critical in psoriasis pathogenesis. Our findings suggest that NF-κB c-Rel plays a critical role in TLR7-mediated inflammation in DCs and warrants consideration of strategies to suppress c-Rel activation as a therapeutic approach in the clinic for psoriasis treatment.

## Methods

### Reagents and antibodies

Recombinant poly (I:C), CpG, imiquimod, CL307, and loxoribine were purchased from Invivogen. Pentoxifylline was purchased from Cayman Chemical. Antibodies against the following proteins were used in this study: c-Rel (D4Y6M and D38BS, Cell Signalling Technology), c-Rel (G7 and B6, Santa Cruz Biotechnology), p65 (F6 and C20, Santa Cruz Biotechnology), p65 (L8F6, Cell Signalling Technology), p50 (D4P4D, Cell Signalling Technology), Sp1 (E3, Santa Cruz Biotechnology), hnRNPA1 (9H10, Santa Cruz Biotechnology), tubulin (H300, Santa Cruz Biotechnology), actin (C4, Santa Cruz Biotechnology), TLR7 (3269, ProSci), PLCy1 (1249, Santa Cruz Biotechnology), lamin A/C (H-110, Santa Cruz Biotechnology), HDAC2 (680104, BioLegend), vinculin (938402, BioLegend), RelB (C19, Santa Cruz Biotechnology), p52 (4882, Cell Signalling Technology). Further details, including catalogue numbers and RRIDs can be found in the supplementary reagent list document. Antibodies used were validated with Western blot analysis using knockout cell lines. Cell suspensions were stained with antibodies against one or more of the following proteins for flow cytometric analysis: CD11c (N418, BioLegend), IL-6 (MP5-20F3, BioLegend), IL-1β (17-7114-80, Thermo Fisher Scientific), CD80 (16-10A1, BioLegend), CD86 (Gl-1, BioLegend), CD4 (GK1.5, BioLegend), IL17A (TC11-18H10.1, BioLegend), Zombie NIR Fixable Viability Kit (BioLegend). Lipofectamine 3000 was purchased from Thermo Fisher and used according to manufacturer’s protocol.

### Cell culture

DC2.4 cells (RRID:CVCL_J409) were cultured in RPMI 1640 medium containing 10% SCS, 1% HEPES, 1% nonessential amino acids, 1% sodium pyruvate, 100 U/mL penicillin/streptomycin, 1% L-glutamine. HaCaT cells (RRID:CVCL_0038) were grown in RPMI 1640 medium containing 10% FBS, 100 U/mL penicillin/streptomycin, and 1% L-glutamine. J558L cells (RRID:CVCL_3949) were grown in DMEM medium containing 10% FBS, 100 U/mL penicillin/streptomycin, 1% L-glutamine for generation of J558L cultured supernatants. Primary bone marrow derived dendritic cells were cultured with 10 mL of RPMI 1640 medium containing 10% FBS, 1% HEPES, 1% nonessential amino acids, 1% sodium pyruvate, 100 U/mL penicillin/streptomycin, 1% L-glutamine, 0.05 mM β-mercaptoethanol with the addition of 1:15 J558L supernatant. All cells were incubated at 37 °C with 5% CO_2_. Cells were routinely tested for mycoplasma contamination and only mycoplasma negative cells were used in all the experiments.

### Generation of CRISPR/Cas9 mediated knockout cells

The CRISPR/Cas9-mediated knockdown of c-Rel, p65, and p50 in DC2.4 cells and HaCaT cells was performed as previously described.^30^^,^^31^ Wild-type DC2.4 cells or HaCaT cells were incubated with lentiviral supernatants expressing vector control or gene specific guide RNA with 8 μg/mL polybrene transfection reagent for 24 h. Cells were selected with 350 μg/mL of hygromycin or 1 μg/mL of puromycin and single cell clone populations were generated, expanded, and tested by Western blotting for expression of the protein of interest. Three or more individual clones of control vector or specific guide RNA infected cells with undetectable amounts of specified protein were pooled and used as controls (designated as WT) or stable knockout (c-Rel KO, p65 KO and p50 KO) cell lines. Guide RNA for c-Rel was used as described previously.^31^ Other guide RNAs used were as follows: p65 - For: CACCGAGCGCCCCTCGCATTTATAG, Rev: AAACCTATAAATGCGAGGGGCGCTC; p50 – For: CACCGACGATGATCCCTACGGAACT, Rev: AAACAGTTCCGTAGGGATCATCGTC.

### Ethics

All mice were handled in accordance with the National Institutes of Health (NIH) guidelines under protocol (#2013-0134) approved by the Case Western Reserve University’s Institutional Animal Care and Use Committee. All the experiments were performed in compliance with animal use guidelines (including the ARRIVE guidelines) and the above ethical approval.

### Mice

The c-Rel knockout mouse line in the C57BL6/J background were kindly provided by H.C. Liou^10^ (Weill Medical College of Cornell University, New York). The C57BL6/J mice were purchased from Jackson Laboratories and maintained in-house. c-Rel KO mice will be available at Jackson Laboratory (Stock No. 039712). Animals were housed in specific-pathogen-free conditions in the animal facility room. Mice had free access to autoclaved food and water. Bedding and nesting materials were provided as standard enrichments to reduce animal distress, and no unexpected adverse events occurred throughout this study. At designated end points, mice were humanely sacrificed in accordance with institutional guidelines using controlled CO_2_ asphyxiation followed by cervical dislocation.

### Imiquimod-induced psoriasis mouse model

For the imiquimod-induced psoriasis, the dorsal skin of mice was shaved, and 62.5 mg of Aldara cream (5% IMQ, 3M Pharmaceuticals) was applied daily on the shaved skin for 5 consecutive days. Mice were weighed daily, photographed, and sacrificed on day 6. Disease severity was assessed daily based on a scoring system based on a modified clinical Psoriasis Area and Severity Index (PASI) as previously described.^24^ Briefly, erythema and scaling were scored in a blinded manner by a dermatologist on a scale from 0 to 4: 0, none; 1, slight; 2, moderate; 3, marked; 4, very marked.

### Quantitative real-time PCR

For DC2.4 cells and BMDCs, total RNA was isolated from cells using EZ10 DNAaway RNA miniprep kit (BioBasic). For skin samples, RNA was purified using TRIzol (Invitrogen). RNA yields were quantified by NanoDrop spectrophotometer, and 1 μg of total RNA was used for cDNA synthesis using High Capacity cDNA Reverse Transcription Kit (Applied Biosystems). Quantitative real-time PCR was performed using PowerUP SYBR Green Master Mix (Applied Biosystems) following manufacturer’s protocol. Primers used to amplify IFNβ were: For: AGATGTCCTCAACTGCTCTC, Rev: AGATTCACTACCAGTCCCAG. Other primer sequences used were previously described.^31^ Samples were normalised to the housekeeping gene L32. Gene expression was quantified using the ΔΔCt method.

### Cell proliferation assay

HaCaT cells (1 × 10^5^/well) were seeded in a 96 well plate 24 h before stimulation. Cell proliferation was measured by Cell Counting Kit-8 (CCK8) (APEx BIO) following manufacturer’s protocol.

### Histology and immunostaining

For haematoxylin and eosin staining, skin samples were processed at the CWRU Skin Diseases Research Center (SDRC) core facility for dermatopathology. Tissue samples were fixed in 4% paraformaldehyde and embedded in paraffin. Deparaffinised 5-μm sections were stained with H&E. Epidermal thickening was assessed by measurements of four representative images from each slide, for each mouse. For immunohistochemistry, 7 μm cryosections were brought to room temperature and fixed with 4% paraformaldehyde for 30 min. Endogenous peroxidase activity was blocked in 0.3% peroxide for 10 min. Sections were blocked and permeabilised in 5% FBS in PBS with 0.5% saponin. Sections were incubated with anti-c-Rel antibody (B6, 1:100, Santa Cruz Biotechnology) overnight at 4 °C. Slides were counterstained with haematoxylin and visualised using an Olympus IX73 microscope.

### Immunoprecipitation and western blotting

Cells were lysed in hypotonic cytoplasmic lysis buffer (10 mM HEPES pH 7.6, 10 mM KCl, 0.1 mM EDTA, 0.1 mM EGTA, 1X protease inhibitor cocktail in dH_2_O) for 15 min on ice. A final concentration of 0.625% NP40 was added to each sample and vortexed to disrupt cellular membranes. Lysates were centrifuged at 12,000 g for 30 s and supernatants were collected as cytoplasmic lysates. Remaining nuclear pellets were washed with hypotonic cytoplasmic lysis buffer 1X. Nuclear pellets were then lysed with hypertonic nuclear lysis buffer (20 mM HEPES pH 7.6, 400 mM NaCl, 1 mM EDTA, 1 mM EGTA, 1X protease inhibitor cocktail in dH_2_O) for 30 min on ice. Lysates were cleared at 12,000 g for 10 min at 4 °C and supernatants were collected following centrifugation. Protein levels were normalised using BCA assay (ThermoFisher Scientific). For immunoprecipitation, cytoplasmic lysates were supplemented with NaCl, and nuclear lysates were diluted with salt free lysis buffer to obtain a final salt concentration of 150 mM in each sample. Lysates were rotated at 4 °C with protein G or protein A beads (Invitrogen) and antibodies against p65, c-Rel or p50 for 3 h. To assess p50 homodimer, samples were subjected to formaldehyde crosslinking^32^ with slight modifications. Following immunoprecipitation, beads were incubated with 4% formaldehyde for 10 min at room temperature. The crosslinking reaction was quenched with the addition of 750 mM Tris (pH 7.5) for 5 min at room temperature. Beads were washed with lysis buffer and heated at 85 °C for 5 min with nonreducing 1X Laemmli sample buffer. For Western blot analysis, lysates were resolved through 9% SDS–PAGE gels. Proteins from the gel were transferred onto nitrocellulose membranes, probed using the indicated antibodies, and visualised by enhanced chemiluminescence assay.

### Oligonucleotide pulldown assay

Oligonucleotide pulldown assay using biotinylated promoter sequences were performed as previously described.^33^ Briefly, nuclear lysates were normalised to 100 μg of total protein and NaCl concentration in the buffer was adjusted to 150 mM. Lysates were incubated with 1 μg of biotinylated oligonucleotides and 6 μg of sheared salmon sperm DNA for 1 h at 4 °C with constant rotation. After 1 h, neutravidin agarose beads (Thermo Scientific) were added and samples were incubated for an additional 2 h. To assess NF-κB complexes bound to the oligonucleotide, beads were incubated with 4% formaldehyde for 10 min at room temperature, and quenched with 750 mM Tris (pH 7.5). Beads were washed with lysis buffer and heated at 85 °C for 5 min with nonreducing 1X Laemmli sample buffer. Samples were analysed by Western blotting. Oligonucleotides containing NF-κB binding sequences were as follows: IL-1β oligonucleotide sequences: forward: CTAACCCAGGAAAACCCAATATT, reverse: AAATATTGGGTTTTCCTGGGTTAG; IL-6 oligonucleotide sequences: forward: CAAATGTGGGATTTTCCCATGAGT, reverse: ACTCATGGGAAAATCCCACATTTG.

### BMDC and T cell coculture

Mouse bone marrow cells were isolated by flushing femurs and tibias with sterile PBS, and strained through a 40 μM filter. All cell suspensions were resuspended in red blood cell lysis buffer (155 mM NH_4_Cl, 12 mM NaCHO_3_, 0.1 mM EDTA in H_2_O) for 5 min. Cells were then plated at 2 × 10^6^/10 cm plate in BMDC RPMI media with added 1:15 J558L conditioned media for GM-CSF supplementation for DC differentiation. Loosely adherent cells were harvested on day 6 and DC differentiation was confirmed by CD11c^+^ flow cytometry. BMDCs (1 × 10^6^/mL) were stimulated with 6 μg/mL of IMQ for 20 h, collected with PBS with 10 mM EDTA, and washed with PBS. Total naïve CD4^+^ T cells were collected from freshly harvested spleens of WT C57BL6/J mice by magnetic sorting with MojoSort CD4 T cell Isolation Kit (480033, BioLegend). Purified T cells were cocultured with BMDCs at a 4:1 ratio in a 96 well plate. For Th17 polarisation conditions, cultures were supplemented with plate bound anti-CD3 (5 μg/mL; 145-2C11, BioLegend) and soluble anti-CD28 (5 μg/mL; 37.51, BioLegend), IL-6 (20 ng/mL; BioLegend), IL-1β (10 ng/mL; BioLegend), TGFβ (2 ng/mL; BioLegend) and the following neutralising antibodies: anti-IL-4 (10 μg/mL; 11B11, BioLegend), anti-IFNγ (10 μg/mL; AN-18, BioLegend), anti-IL2 (10 μg/mL; JES6-1A12, BioLegend). For Th0 polarising conditions, cultures were supplemented with plate bound anti-CD3 (2 μg/mL; 145-2C11, BioLegend) and soluble anti-CD28 (1 μg/mL; 37.51, BioLegend). After 3 days, cocultures were stimulated with PMA, ionomycin, and brefeldin A for 5 h. Cells were harvested and stained with surface antibodies and Zombie NIR Viability Kit for 20 min at 4 °C. For intracellular staining, cells were fixed and permeabilised according to manufacturer’s recommendation (True-Nuclear Transcription Factor Buffer Set, BioLegend) and stained with intracellular antibodies overnight at 4 °C. Samples were analysed with CytoFLEX flow cytometer (Beckman Coulter) and FlowJo software (TreeStar).

### Statistics

Data are presented as means with 95% CI. Shapiro–Wilk test and generated Q–Q plots confirmed normality of all variable data (p > 0.05). Homogeneity of variance was confirmed by examining the homoscedasticity of residual plots. Differences between groups were compared by two-way ANOVA, followed by pairwise comparisons via Tukey’s multiple comparisons when all the pairwise comparisons were of interest or Sidak’s multiple comparison when specific pairwise comparisons were of interest. The factors used for two-way ANOVA were mouse genotype (WT or c-Rel KO) and treatment (ctrl or IMQ). Mixed effects model was used to assess the difference in temporal patterns between groups considering the correlation of observations within experimental units. Time, treatment and genotype correspond to fixed effects while mice correspond to the random effects in this model. We assessed the assumption of sphericity with Geisser-Greenhouse epsilon and applied the corresponding correction if the assumption of sphericity was violated. We assessed the assumption of linearity by examining plots of residuals against predicted values. Two-tailed unpaired Student’s t-test was performed for unmatched groups. Study design typified a clustered comparison with four clusters per group and four randomly selected observations per cluster. Power analysis for *in vivo* studies was performed for epidermal acanthosis measurements,^34^ which estimated that four animals per group were sufficient to obtain a power of 0.8 with a significance p < 0.05.

### Role of funders

The funders had no role in planning the study, in the experimental, data collection, analysis, and interpretation, in the writing of the manuscript, and in the decision to submit the manuscript for publication.

## Results

### NF-κB c-rel expression is upregulated in human and IMQ-induced mouse psoriasis

**Fig. 1:**
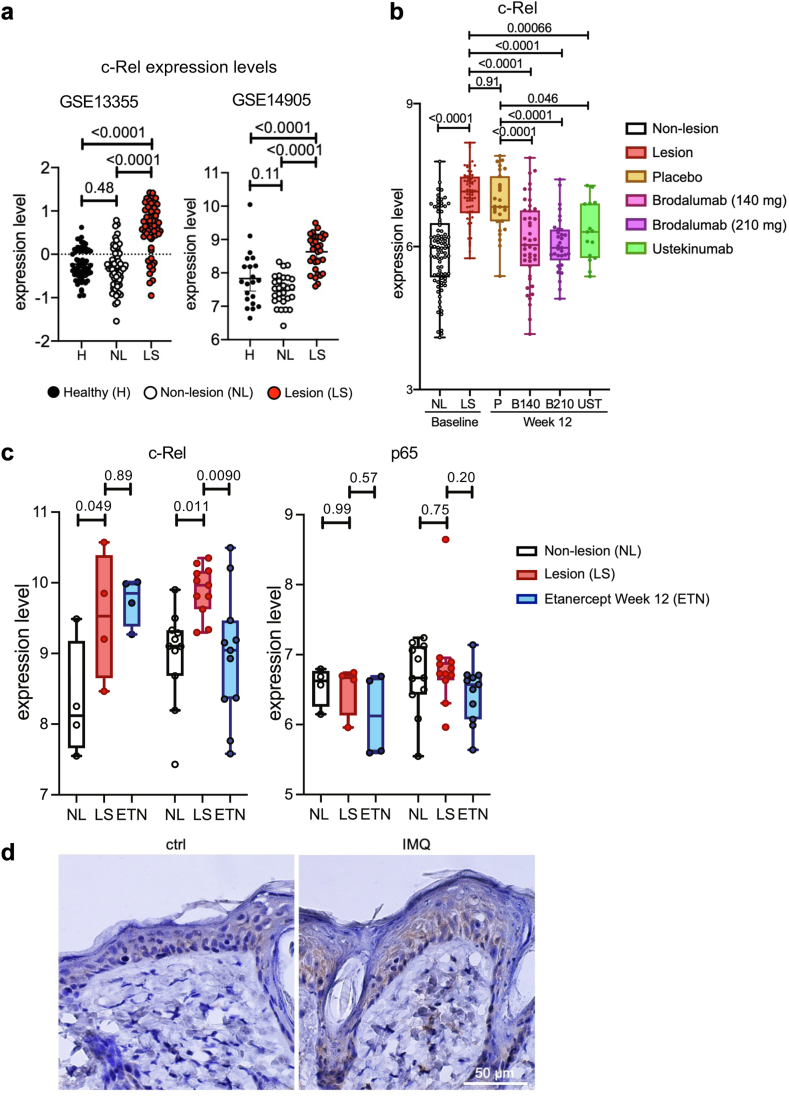
**c-Rel expression is increased in the skin of human patients with psoriasis and IMQ-induced psoriasis-like mouse model.** (a) c-Rel levels in different public GEO datasets of healthy, nonlesional, and psoriatic lesional skin biopsies (GSE13355, GSE14905). (b, c) c-Rel and (c) p65 levels in psoriatic lesions in patients treated with different biologics (GSE117468, GSE11903). Box and whisker plots tails are defined as 1.5 times interquartile range (IQR) from the first and third quartiles of each dataset. (a–c) Each point is representative of mean expression with 95% CI (One-way ANOVA, Tukey’s multiple comparisons test). (d) Representative immunohistochemistry staining of c-Rel in control or IMQ-treated dorsal skin of C57BL/6J mice at day 5, n = 3 (magnification: 60×, scale bar = 50 μm).

To assess whether NF-κB c-Rel may be functionally relevant in psoriasis, we analysed the expression of NF-κB c-Rel in psoriatic skin biopsies from seven transcriptome datasets from the Gene Expression Omnibus (GEO) database (Supplementary Table S1). Compared to healthy and nonlesional skin biopsies, we found a marked increase of c-Rel expression in psoriatic lesional skin in all datasets analysed (Fig. 1a–c and Supplementary Fig. S1).^35^, ^36^, ^37^, ^38^, ^39^ Interestingly, c-Rel expression was downregulated following treatment with brodalumab (GSE117468), ustekinumab (GSE117468)^40^ and etanercept (GSE11903)^41^ (Fig. 1b and c). Of note, the downregulation of c-Rel following etanercept treatment was only seen in patients who successfully responded to the biologic and achieved histological disease resolution, while c-Rel levels remained elevated in non-responders (Fig. 1c). Given the amount of structural and functional overlap that can exist between NF-κB subunits,^42^ we also checked the expression of NF-κB p65 in these samples. In contrast to c-Rel, there are no substantial changes in the levels of p65 among non-lesional, lesional, and etanercept treated skin biopsies in both non-responder and responder patients (Fig. 1c). Consistent with our transcriptomics analysis, c-Rel levels were increased in the dorsal skin of IMQ-induced psoriasis mouse model, specifically in the dermis (Fig. 1d and Supplementary Fig. S2). Taken together, data from both human and mouse models indicate that c-Rel expression is increased in psoriatic lesional skin.

### c-Rel deficiency protects mice from IMQ-induced psoriasis

**Fig. 2:**
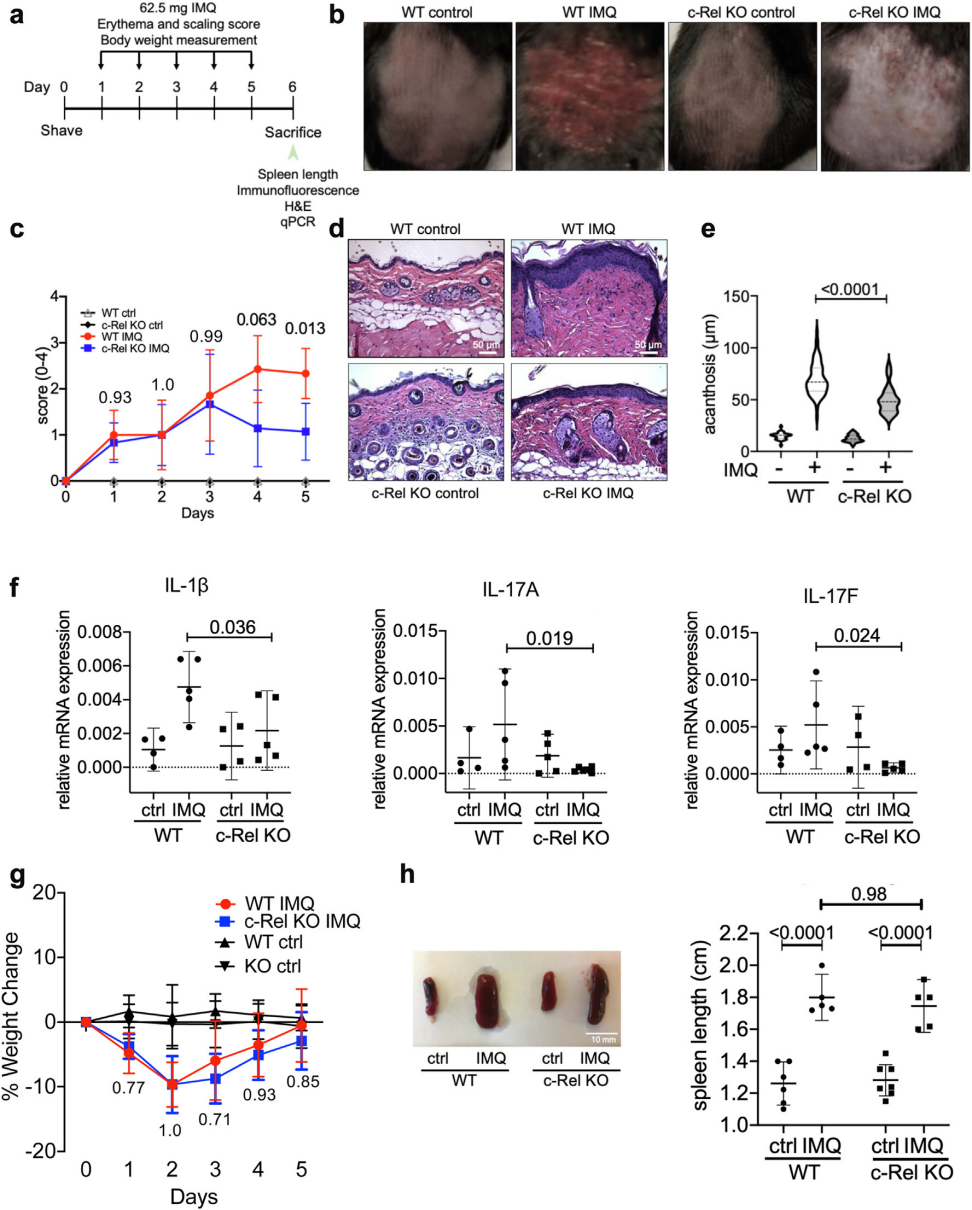
**c-Rel deficiency protects mice from TLR7-induced psoriasiform skin pathology but not from systemic inflammation.** (a) Schematic of daily topical application of IMQ to the dorsal skin of WT and c-Rel KO mice. (b) Representative images of the dorsal areas of WT and c-Rel KO mice following 5 d IMQ treatment or controls (actual unscaled photograph). (c) Daily scores of erythema and scaling of the shaved dorsal skin on a scale from 0 to 4. The assumption of sphericity was violated (Geisser-Greenhouse epsilon = 0.62) and the Geisser-Greenhouse correction was applied. Each point is representative of the mean score with 95% CI (Two-way ANOVA, mixed-effects analysis with Tukey’s multiple comparisons test, n = 6). (d) Histological sections of the dorsal skin were stained with H&E for acanthosis measurement (n = 4, scale bar = 50 μm). (e) Average thickness of four separate measurements were taken from four different locations in the epidermis of each skin section (magnification: 20×) (Two-way ANOVA, Sidak’s multiple comparisons test, n = 4). (f) Expression of IL-1β, IL-17A, and IL-17F in the dorsal skin sections of control and IMQ treated WT and c-Rel KO mice relative to the housekeeping gene L32. Each data point is the mean of technical duplicates per mouse and represented as means with 95% CI (Two-way ANOVA, Sidak’s multiple comparisons test, n = 4–5). (g) Daily body weight changes of control and IMQ treated WT and c-Rel KO mice represented as means with 95% CI (Two-way ANOVA, mixed-effects analysis with Tukey’s multiple comparisons test, n = 6). (h) Representative spleens of indicated mice (left, scale bar = 10 mm) and their lengths (right) were measured and represented as means with 95% CI (Two-way ANOVA, Sidak’s multiple comparisons test, n = 6).

To further confirm that c-Rel is relevant to psoriasis, we utilised an IMQ-induced psoriasis-like mouse model^24^ using WT and c-Rel KO (C57BL/6J) mice. WT and c-Rel KO mice were shaved 1 day prior to application of 62.5 mg IMQ cream daily to the dorsal skin for five days (Fig. 2a). Treatment with IMQ induced a psoriasiform skin pathology, which was reduced in c-Rel KO C57BL/6J mice (Fig. 2b). Erythema and scaling were scored daily, with all mice starting with a score of 0. c-Rel KO mice had a lower erythema and scaling score starting at Day 4 as measured by raw score (Fig. 2c) and standard mean difference (Supplementary Fig. S2b). To observe histological changes, dorsal skin biopsies were taken on Day 6 and epidermal thickness was measured. c-Rel KO mice exhibited substantially reduced IMQ-induced acanthosis of their dorsal skin compared to WT mice. However, compared to untreated controls, c-Rel KO mice still showed modestly enhanced epidermal thickness, suggesting residual inflammation in the absence of c-Rel (Fig. 2d and e). We examined expression of inflammatory genes in the dorsal skin of untreated and IMQ-treated WT and c-Rel KO mice and found increased expression of IL-1β, IL-17A, and IL-17F in WT IMQ-treated mice compared to c-Rel KO IMQ-treated mice (Fig. 2f). These findings suggest that the c-Rel is critical in promoting the TLR7-induced psoriasiform skin pathology.

Topical IMQ treatment has been shown to cause systemic effects in mice including loss of body weight and immune activation.^24^^,^^43^ Interestingly, c-Rel deficiency did not prevent overall weight loss, as WT and c-Rel KO mice showed similar systemic body weight loss in response to IMQ-treatment (Fig. 2g). We also found that both WT and c-Rel KO mice showed comparable splenomegaly as an indicator of immune activation (Fig. 2h). This suggests that c-Rel may regulate IMQ-induced localised skin inflammation, and is not critical for the systemic inflammation, which can be caused by IMQ treatment.

### NF-κB c-rel deficiency inhibits TLR7-mediated inflammation in dendritic cells

The IMQ-mouse model agonises TLR7 to cause a psoriasis-like phenotype^24^ and TLR7 has been suggested to be critical for the inflammation which occurs in psoriasis.^44^ Our analysis of psoriatic skin biopsies from transcriptome datasets also found the upregulation of NF-κB c-Rel, but not NF-κB p65, in psoriatic lesions. Thus, we hypothesised that the TLR7/c-Rel axis may be a signalling mechanism involved in psoriasis pathogenesis. Immunohistochemistry staining indicated that c-Rel was increased in both the epidermis and the dermis layer of the skin (Fig. 1d), suggesting that c-Rel function in keratinocytes in the epidermis or immune cells that infiltrated into the dermis may contribute to the TLR7-induced inflammation. Psoriasis causes an infiltration of immune cells, primarily T cells and DCs, to the dermis which causes keratinocyte hyperplasia leading to the inflamed psoriatic lesion.^3^ c-Rel has been found to be critical in keratinocyte proliferation.^45^^,^^46^ We first evaluated TLR7 signalling on keratinocytes by using the spontaneously transformed human keratinocyte cell line, HaCaT.^47^ IMQ was able to induce NF-κB translocation by 1 h in HaCaT cells (Supplementary Fig. S3a and b), indicating active TLR7 signalling in these cells. We then generated HaCaT cells that lack c-Rel using CRISPR-Cas9-mediated gene editing. The expression of TLR7 in keratinocytes is debated.^48^, ^49^, ^50^ We found that WT HaCaT cells expressed TLR7 and the deletion of c-Rel did not affect TLR7 expression in these cells (Supplementary Fig. S3c). Next, we studied whether the genetic absence of c-Rel results in altered TLR7-induced inflammatory gene expression. IL-1β and IL-6 are commonly upregulated inflammatory cytokines during psoriasis and stimulation of keratinocytes with TLR agonists have been shown to induce their expression.^51^ We found that IMQ stimulation induced similar levels of IL-1β and IL-6 expression in both WT and c-Rel KO HaCaTs (Supplementary Fig. S3d). We also studied IMQ-induced proliferation of WT and c-Rel KO HaCaT cells. We found that IMQ stimulation had no effect on the proliferation of both WT and c-Rel KO HaCaT cells (Supplementary Fig. S3e) and the absence of c-Rel did not alter keratinocyte proliferation (Supplementary Fig. S3e). Taken together, these results suggest that TLR7 signalling does not affect keratinocyte proliferation and c-Rel is dispensable for the expression of psoriasis relevant inflammatory cytokines in HaCaT cells. Hence, we hypothesised that the cellular mechanism controlled by TLR7/c-Rel axis in psoriasis pathogenesis was likely not through its cell intrinsic function in keratinocytes.

**Fig. 3:**
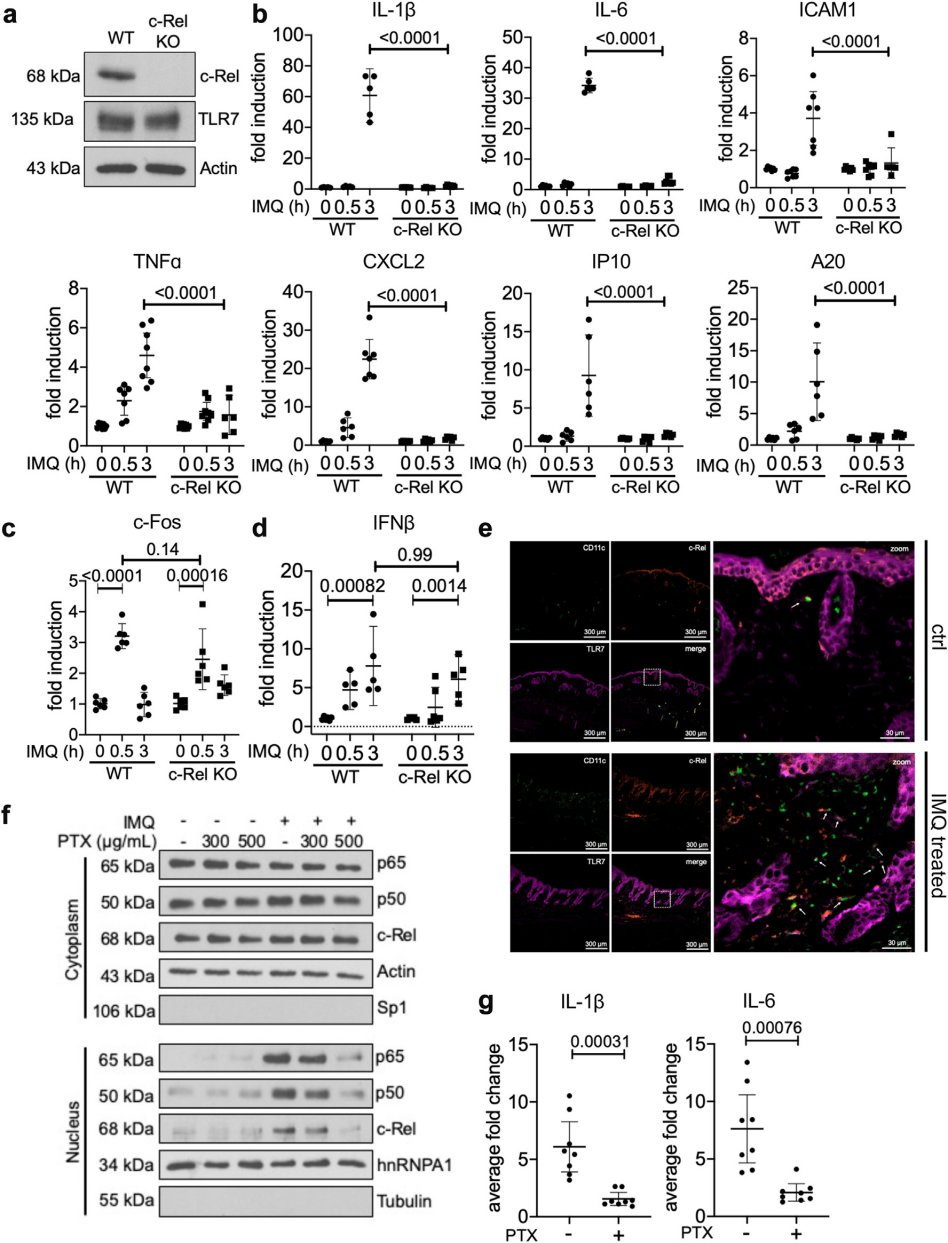
**c-Rel is critical for TLR7-induced proinflammatory gene expression in DC2.4 cells.** (a) Total lysates of WT and c-Rel KO DC2.4 cells were analysed by Western blotting for TLR7 and c-Rel expression in WT and c-Rel KO DC2.4 cells. Actin was used as a loading control. (b–d) WT and c-Rel KO DC2.4 cells were stimulated with IMQ for 0 h, 0.5 h and 3 h. Expression of proinflammatory genes (b) IL-1β, IL-6, ICAM1, TNFα, CXCL2, IP10, A20, (c) c-Fos and (d) IFNβ were determined by quantitative real-time PCR. Data in (b–d) are shown as technical duplicates from at least three separate experiments and represented as means with 95% CI (Two-way ANOVA, Sidak’s multiple comparisons test). (e) Dorsal skin sections from control and IMQ treated WT C57BL/6 mice were stained for CD11c, c-Rel and TLR7 (n = 4, scale bar = 300 μm, zoom scale bar = 30 μm). (f) WT DC2.4 cells were pretreated with indicated amounts of PTX for 0.5 h. IMQ was added as indicated for an additional 0.5 h. Cytoplasmic and nuclear extracts were prepared and analysed by Western blotting with antibodies for p65, p50, and c-Rel. Actin and hnRNPA1 were used as loading controls and Sp1 and tubulin were used as purity controls for cytoplasmic and nuclear fractions, respectively. (g) WT DC2.4 cells were pretreated with 500 μg/mL PTX for 0.5 h then treated with IMQ for an additional 3 h. Data are represented as the average fold change over cells not treated with IMQ and plotted as technical duplicates from four separate experiments (two-tailed unpaired Student’s t test). All values were normalised to the housekeeping gene L32, and expression of target genes was determined using the ΔΔCt method.

TLR7 is predominantly expressed in DCs^20^, ^21^, ^22^ and NF-κB c-Rel is also abundantly expressed in DCs.^52^ To study the role of c-Rel in TLR7 signalling in dendritic cells, we used CRISPR-Cas9 mediated gene editing to generate c-Rel deficient immortalised dendritic cells (DC2.4s) (Fig. 3a). Our previous work showed that c-Rel can act as a repressor of inflammatory gene expression and can also change the kinetics of gene expression.^31^ Hence, we studied TLR7-induced inflammatory gene expression at 30 min and 3 h in DC2.4 cells, to determine whether the genetic absence of c-Rel results in altered inflammatory gene expression. We found that the transcription of IL-1β and IL-6 was induced in WT DC2.4 cells following IMQ stimulation, but not in c-Rel deficient cells (Fig. 3b). We further found that the transcription of other inflammatory genes examined (ICAM1, TNFα, CXCL2, IP10, A20) were also induced at 3 h in WT cells but not in c-Rel deficient cells (Fig. 3b). ICAM1 and TNFα showed a 4-fold reduction in expression while several other genes (IL-1β, IL-6, CXCL2, IP10, and A20) were reduced more than 10-fold in c-Rel deficient cells when compared to WT cells (Fig. 3b). To exclude the possibility that absence of c-Rel compromises general transcription, we checked the expression of an NF-κB independent gene, c-Fos, and found that its expression was not dependent on c-Rel (Fig. 3c). IMQ stimulation was able to induce similar levels of c-Fos expression by 30 min in both WT and c-Rel deficient cells (Fig. 3c). We also assessed the expression of the type 1 interferon, IFNβ, which is induced by TLR7 in DCs^53^ but has been reported to be regulated by the noncanonical NF-κB pathway.^54^ We found that IMQ-induced expression of IFNβ expression was not affected in c-Rel deficient DCs (Fig. 3d). Together, these findings suggest that c-Rel is required for the induction of select inflammatory cytokine and chemokine genes following TLR7 stimulation in DCs. This is likely due to the role of c-Rel as a transcription factor in TLR7 signalling, and not due to a difference in the expression of TLR7, as WT and c-Rel KO DC2.4s had comparable amounts of TLR7 (Fig. 3a and Supplementary Fig. S4). Further confirming that this signalling axis is relevant in DCs during psoriasis *in vivo*, we found a substantial increase in the colocalisation of TLR7, c-Rel, and CD11c in the dermis of IMQ-treated dorsal skin samples of WT mice (Fig. 3e). The importance of c-Rel and TLR7 have separately been described in DCs,^11^^,^^12^^,^^22^ however, the TLR7/c-Rel signalling axis has not yet been described. Taken together, these data suggest that c-Rel has a critical transcriptional regulatory role in TLR7-induced inflammatory signalling in DCs during psoriasis.

In addition to the genetic c-Rel KO model, we sought to chemically inhibit c-Rel to further validate the role of c-Rel in TLR7 signalling. We used Pentoxifylline (PTX), a xanthine derivative that has previously been shown to be a potent inhibitor of c-Rel function.^55^^,^^56^ Treatment of DC2.4s with 300 μg/mL of PTX resulted in a slight decrease and 500 μg/mL PTX resulted in a more substantial decrease in the nuclear translocation of NF-κB c-Rel, p65, and p50 (Fig. 3f). Treatment with 500 μg/mL PTX also inhibited the production of IL-6 and IL-1β following stimulation with IMQ (Fig. 3g). Taken together, this suggested that chemical inhibition of NF-κB resulted in the inhibition of TLR7-mediated inflammatory signalling in DCs.

### NF-κB c-rel regulates inflammatory cytokine expression in dendritic cells following multiple chemically distinct TLR7, but not TLR3 or TLR9, agonist treatments

**Fig. 4:**
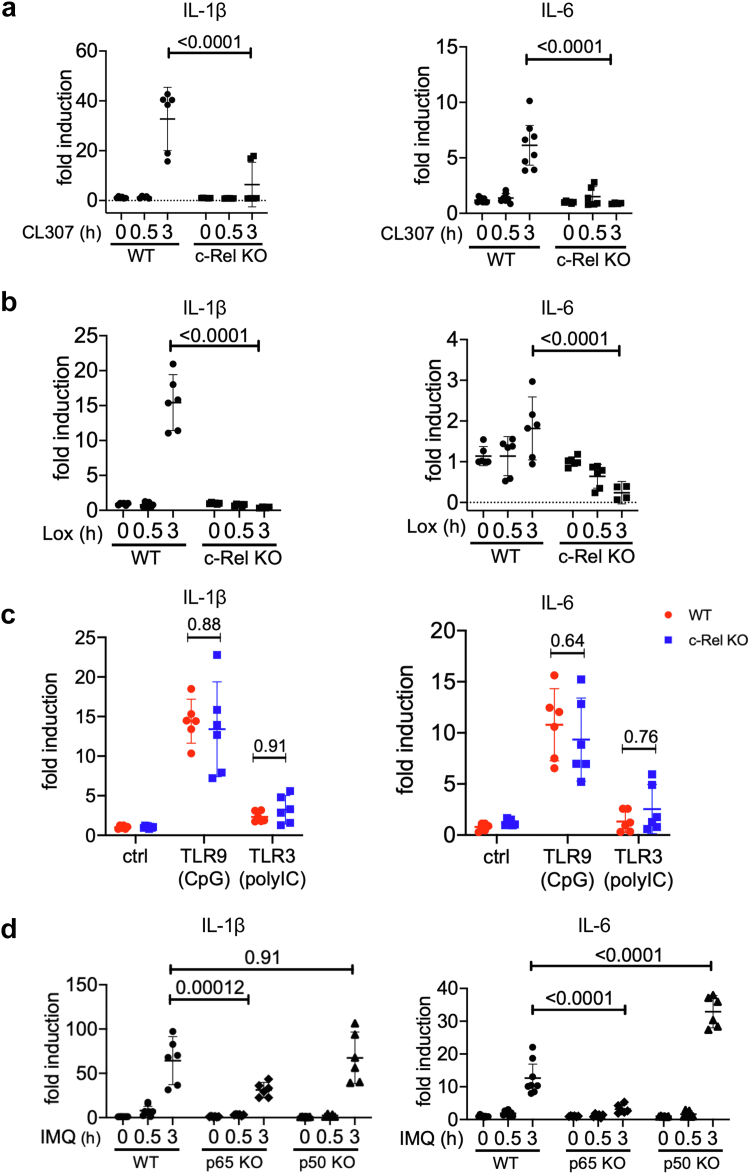
**c-Rel is critical for proinflammatory gene expression following TLR7, but not TLR3 or TLR9 agonists and both c-Rel and p65 are required for IMQ-induced inflammatory gene expression.** (a and b) WT and c-Rel KO DC2.4 cells were treated with (a) CL307 (200 ng/mL) and (b) loxoribine (2 mM) for 0 h, 0.5 h, and 3 h. (c) WT and c-Rel KO DC2.4 cells were stimulated with CpG (1 μM) or poly (I:C) (12.5 μg/mL) for 3 h. (d) WT, p65 KO, and p50 KO DC2.4 cells were treated with IMQ for 0 h, 0.5 h, and 3 h. (a–d) Gene expression of IL-1β and IL-6 were determined by quantitative real-time PCR using the ΔΔCt method. All values were normalised to the housekeeping gene L32. Data are shown as technical duplicates from at least three separate experiments and represented as means with 95% CI (Two-way ANOVA, (a–c) Sidak’s multiple comparisons test, and (d) Tukey’s multiple comparisons test).

To ensure that the IMQ-induced c-Rel dependent inflammatory signalling was exclusively due to effects of TLR7 stimulation and signalling, and not due to any nonspecific effect of IMQ, we treated our WT and c-Rel deficient DC2.4 cells with two other chemically distinct TLR7 agonists, CL307 and loxoribine. CL307 is an adenine analogue^57^ while loxoribine is a guanine derivative^58^ that both exclusively signal via TLR7. Stimulation of WT DC2.4 cells with CL307 and loxoribine resulted in a robust increase of IL-1β expression, while IL-6 expression was induced only modestly. Nevertheless, absence of c-Rel compromised both CL307-and loxoribine-induced increase in IL-1β and IL-6 production (Fig. 4a and b). Given the robust effect on DCs, clinical relevance, and similar results as other TLR7 agonists, we chose to use IMQ in subsequent experiments.

TLR7 is located in the endosome and structurally homologous with other TLRs, such as TLR9.^58^ To study the specific regulatory role of c-Rel in endosomal TLR signalling, we stimulated WT and c-Rel KO DC2.4s with CpG and poly (I:C) to stimulate TLR9 and TLR3, respectively. We found that CpG was able to induce a substantial increase in the production of IL-1β and IL-6 in wild-type DC2.4s, while poly (I:C) resulted in only a modest induction (Fig. 4c). Notably, c-Rel deficiency did not affect the transcription of inflammatory cytokines after 3 h of TLR9 or TLR3 stimulation (Fig. 4c). This suggests that TLR7 has a distinct signalling mechanism from TLR3 and TLR9, and that c-Rel is uniquely necessary for TLR7-induced proinflammatory signalling in DCs.

### NF-κB p65, but not p50, deficiency decreases TLR7-induced inflammatory cytokine expression in dendritic cells

NF-κB subunits are structurally similar via a shared Rel-homology domain and display both unique and redundant functional roles.^42^ To study the unique and redundant role of c-Rel in TLR7 signalling with NF-κB p65 and NF-κB p50, the other subunits within the canonical pathway of NF-κB signalling, we generated p65 and p50 (Supplementary Fig. S5a and b) deficient DC2.4s using CRISPR-Cas9-mediated gene editing. We observed that p65 deficient DC2.4s were also unable to produce high levels of inflammatory cytokines following TLR7 stimulation (Fig. 4d). This confirms previous studies showing that p65 inhibition ameliorates TLR7-induced inflammation.^59^, ^60^, ^61^ TLR7 stimulation of p50-deficient DC2.4s resulted in comparable amounts of IL-1β production as WT cells (Fig. 4d). This is in line with previous studies that have found that IL-1β production in activated immune cells is not mediated by p50.^62^ In contrast, p50-deficient DC2.4s produced an increased amount of IL-6 compared to WT cells following TLR7 stimulation (Fig. 4d). The inability of both c-Rel KO and p65 KO cells to express IL-1β and IL-6 following TLR7 stimulation suggests that both c-Rel and p65 is required in this signalling pathway. Taken together, this suggests that an activating p65/c-Rel heterodimer is being formed following TLR7 stimulation, which may be the key NF-κB dimer regulating TLR7-induced inflammatory cytokine expression.

### c-Rel knockout enhances TLR7-induced nuclear translocation of p65 and the translocation and DNA binding of p50

NF-κB-dependent gene transcription involves signal induced nuclear translocation of homo- and heterodimers of NF-κB proteins and their binding to cognate promoter sequences. Mechanistic studies of the specific role of c-Rel in TLR7 and how it interacts with other canonical subunits of NF-κB during TLR7-induced inflammation have not been previously described. We assessed IMQ-induced nuclear translocation of NF-κB subunits in WT and c-Rel KO DC2.4s to determine the altered inflammatory cytokine production in c-Rel KO cells as a function of specific NF-κB subunits in the nucleus. We found that c-Rel KO cells had a basal increase of p65, p50, RelB, and p100 in the nucleus, and a basal increase of p50, RelB and p100 in the cytoplasm (Supplementary Fig. S6a–c). This is likely due to the functional compensation that exists between NF-κB dimers.^42^ RelB can act as both a transcriptional activator and participate in inhibitory complexes which repress NF-κB activity.^63^ Hence, we investigated the possibility that increased levels of RelB may act as a suppressor of IL-1β and IL-6 transcription in c-Rel deficient cells. We overexpressed RelB in WT DC2.4s (Supplementary Fig. S6d) and found that an increase in RelB did not inhibit IMQ-induced IL-1β and IL-6 gene expression (Supplementary Fig. S6e). This suggests that RelB, in spite of the enhanced basal expression level, may not constitute an inhibitory dimer in c-Rel KO DCs.

**Fig. 5:**
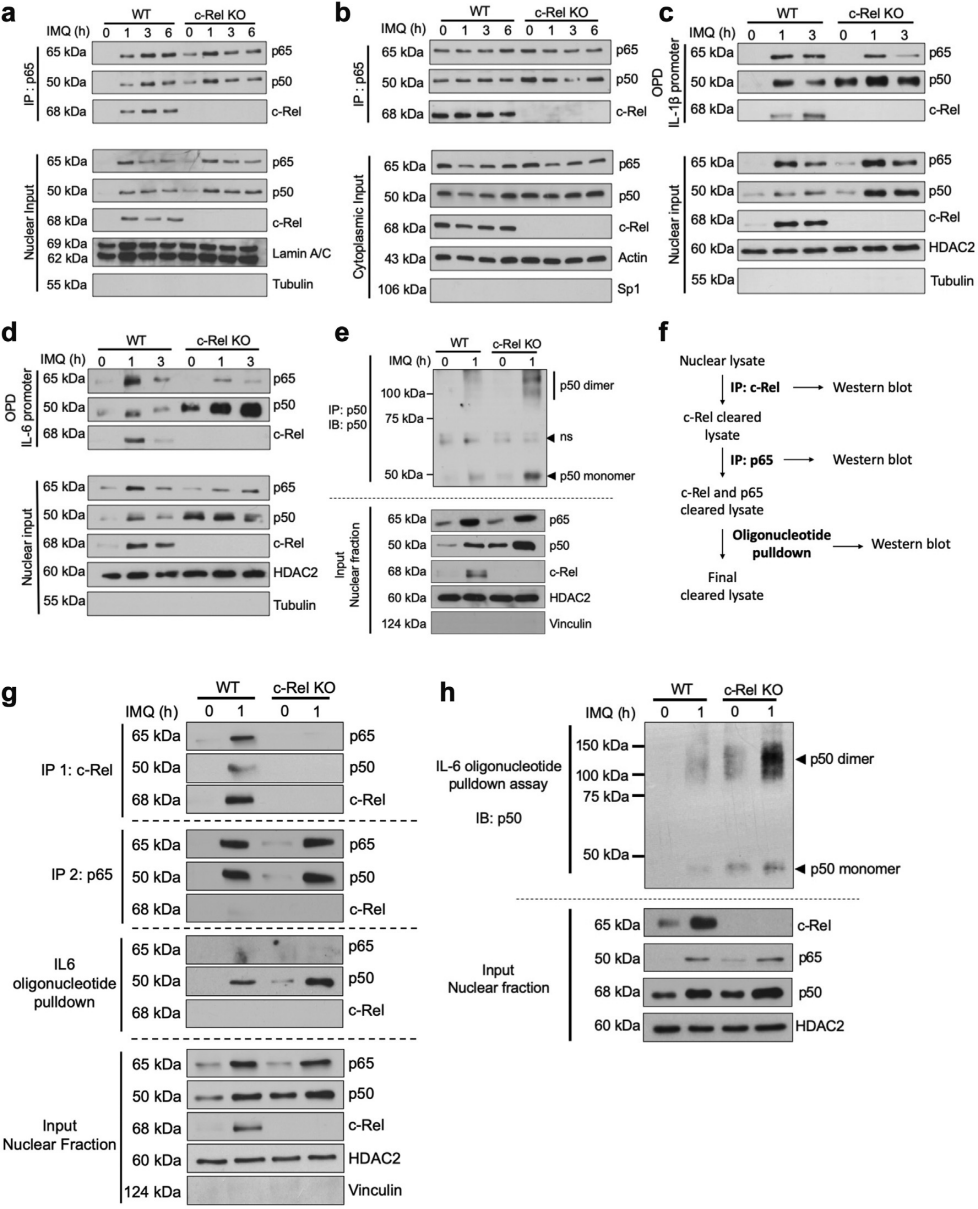
**c-Rel deficiency enriches p50 containing NF-κB dimers binding to IL-1β and IL-6 promoters.** (a–h) WT and c-Rel KO DC2.4 cells were stimulated with IMQ for the indicated time points. Nuclear and cytoplasmic fractions were prepared. (a, b) Nuclear and cytoplasmic extracts subjected to immunoprecipitation (IP) with an anti-p65 antibody (top panels), and lysate input for the amounts of respective proteins in each sample (bottom panels) were analysed by Western blotting (IB) with antibodies against indicated proteins. Lamin A/C and actin were used as loading controls and tubulin and Sp1 were used as the nuclear and cytoplasmic fraction purity controls, respectively. (c and d, g and h). Top: 100 μg of the nuclear fractions were utilised in a pull-down assay using biotinylated (c) IL-1β promoter oligonucleotide or (d, g-h) IL-6 promoter oligonucleotide. (c–e, g and h) Bottom: Amounts of indicated proteins in the nuclear extracts. HDAC2 was used as a loading control. Tubulin or vinculin were used as a nuclear fraction purity control. (e) Nuclear fractions were subjected to immunoprecipitation (IP) with an anti-p50 antibody. Protein complexes were crosslinked in 4% formaldehyde before analysis with Western blotting (IB) with antibody against p50. (f) Schematic of workflow for sequential immunoprecipitation. (g) Sequential immunoprecipitation with indicated antibodies was performed on 100 μg of nuclear lysates. Three rounds of immunoprecipitation with an anti-p65 antibody was performed before proceeding to oligonucleotide pulldown assay with biotinylated IL-6 promoter oligo. (h) Nuclear fraction was used in an oligonucleotide pulldown assay using biotinylated IL-6 oligonucleotide. Samples were crosslinked in 4% formaldehyde before analysis with Western blotting (IB) with antibody against p50. (a–e, g and h) Data is representative of three independent experiments.

To further dissect the molecular mechanism of c-Rel in TLR7 signalling, we performed a kinetic analysis by stimulating WT and c-Rel deficient DC2.4s with IMQ at timepoints from 1 h through 6 h and assessed nuclear and cytoplasmic fractions by western blotting. We found a time-dependent translocation into and out of the nucleus of c-Rel, p65, and p50 where their amounts were increased in WT cells starting at 1 h, and decreased by 3 h. Similarly, p65 and p50 translocated into the nucleus of WT and c-Rel KO DC2.4s at 1 h. Interestingly, p50 translocated to the nucleus starting at 1 h and was sustained until 6 h in c-Rel KO DC2.4s (Fig. 5a bottom, Supplementary Fig. S6a and b). p65 nuclear translocation at 3 h and 6 h was not enhanced in c-Rel KO DC2.4s as compared to WT cells (Fig. 5a, bottom, Supplementary Fig. S6a and b). In the absence of c-Rel, it is possible that the presence of nuclear p50 may contribute to a greater ratio of p65/p50 heterodimers. This suggests that although p65/p50 heterodimers are typically thought of as activating dimers,^64^ at least in c-Rel deficient DCs, the sustained translocation of p50 is not involved in forming dimers that activate the transcription of the proinflammatory genes described here.

Next, we investigated whether the sustained nuclear translocation of the p50 subunit seen in our c-Rel-deficient cells exists as homo- or heterodimers. The p65/p50 heterodimer is abundant within the canonical NF-κB pathway and has been described to activate the transcription of inflammatory cytokines.^64^, ^65^, ^66^ On the other hand, the p50 homodimer has been described to inhibit the transcription of inflammatory cytokines.^66^, ^67^, ^68^, ^69^, ^70^ In part, this is because p50 lacks a transactivation domain and thus binding to DNA blocks access to other NF-κB dimers.^71^ To examine p65/p50 heterodimers, we immunoprecipitated NF-κB p65 from the cytoplasmic and nuclear fractions of wild-type and c-Rel deficient cells after 0 h, 1 h, 3 h, and 6 h of IMQ stimulation. We found similar amounts of p65/c-Rel and p65/p50 heterodimers present in the cytoplasm of WT cells at the time points indicated (Fig. 5b, top). Corresponding to the basal increase in p65 and p50 subunits seen in the cytoplasmic fraction of c-Rel KO DC2.4s, we found a basal increase in the amount of p65/p50 heterodimer found in the cytoplasm of c-Rel KO cells, potentially due to the replacement of p65/c-Rel dimers with p65/p50 dimers in the absence of c-Rel (Fig. 5b, top). Of note, there was also an increase in the basal level of the p65/p50 heterodimer in the nucleus of c-Rel deficient cells at the 0 h time point as compared to the WT cells (Fig. 5a, top). However, when comparing WT and c-Rel deficient DCs after 3 h and 6 h of IMQ stimulation, no enhancement of the p65/p50 dimer in the nucleus was observed, showing that there is an overall decrease of the p65/p50 heterodimer translocated to the nucleus following TLR7 stimulation of c-Rel KO cells (Fig. 5a, top, lane 3, 4, 7, 8). Together, these observations suggest that although there is enhanced translocation of p50 in the c-Rel deficient cells, this subunit is not forming p65/p50 heterodimers at an increased ratio as compared to WT cells. Therefore, we hypothesised that the increased levels of p50 found in the nucleus of c-Rel deficient cells forms an inhibitory p50 homodimer, thereby suppressing the production of inflammatory cytokines and chemokines.

Effective DNA binding of the translocated NF-κB dimers should accompany nuclear translocation to regulate transcription. To study the specific TLR7-induced DNA binding NF-κB subunits in WT and c-Rel KO cells, we designed biotinylated oligonucleotides of the promoter regions of IL-1β and IL-6 and performed an oligonucleotide pulldown assay. We found that p65 binding to the IL-1β promoter was decreased in the nuclear extracts of c-Rel deficient DC2.4 cells at 3 h compared to the WT cells. In contrast, there was sustained binding of p50 to the IL-1β promoter at 1 and 3 h (Fig. 5c, top), further suggesting that the p50/p50 homodimer may be causing the inhibition of IL-1β transcription in c-Rel deficient cells. Similarly, we found decreased p65 binding to the IL-6 promoter at 1 and 3 h (Fig. 5d, top). Strikingly, there is a substantially increased amount of p50 bound to the IL-6 promoter at 1 and 3 h (Fig. 5d), further suggesting that a p50/p50 homodimer inhibits the transcription of IL-6 in c-Rel deficient cells.

To investigate whether increased levels of p50 in IMQ-stimulated c-Rel deficient cells form dimers, we immunoprecipitated p50 from nuclear fractions after 0 h and 1 h of IMQ stimulation in WT and c-Rel KO DC2.4s and crosslinked samples to preserve native conformations (Fig. 5e). We found an increase of p50 containing high molecular weight complexes present in IMQ-stimulated c-Rel deficient cells as compared to WT cells. To confirm that these complexes indeed contain p50/p50 homodimer, we performed sequential immunoprecipitation to deplete c-Rel and p65 containing complexes from the nuclear lysate (Fig. 5f). We assessed the depleted lysate to determine if the increased formation of the p50/p50 homodimer in stimulated c-Rel KO cells was functional in binding to the IL-6 promoter DNA, which would lead to the inhibition of IL-6 transcription. We found a substantial enrichment of p50, with no signal for p65 or c-Rel, indicating the induction of a p50 homodimer following TLR7 stimulation in the absence of c-Rel (Fig. 5g). Crosslinking samples following the oligonucleotide pulldown, showed an enriched p50 complex bound to IL-6 DNA around 100 kDa, further suggesting the formation of a p50/p50 homodimer in the absence of c-Rel (Fig. 5h).

### c-Rel is critical in transcriptional regulation of TLR-mediated inflammatory cytokines in primary dendritic cells

**Fig. 6:**
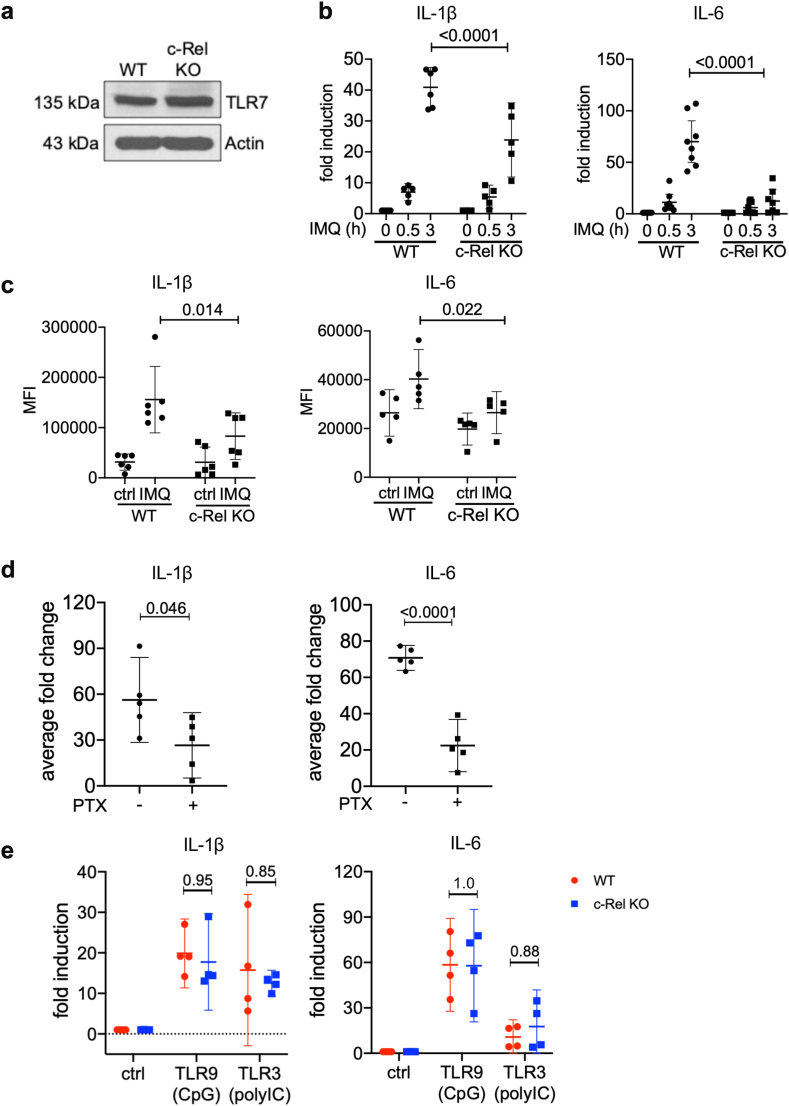
**c-Rel is critical for the production of proinflammatory genes in primary BMDCs.** (a) Total lysates of WT and c-Rel KO BMDCs were probed to assess TLR7 expression. Actin was used as a loading control. (b) WT and c-Rel KO BMDCs were collected and stimulated with IMQ for 0 h, 0.5 h, 3 h and analysed for the expression of IL-1β and IL-6. (c) WT and c-Rel KO BMDCs were stimulated with IMQ for 8 h and assessed for protein expression of IL-1β and IL-6 via intracellular flow cytometry. (d) WT and c-Rel KO BMDCs were pretreated for 0.5 h with 500 μg/mL PTX and then treated with IMQ for an additional 3 h. Data are represented as the average fold change over cells not treated with IMQ. (e) WT and c-Rel KO BMDCs were stimulated with CpG (1 μM) and poly (I:C) (12.5 μg/mL) for 3 h. (b–e) Expression of target genes were determined by quantitative real-time PCR using the ΔΔCt method. Each data point is representative of the mean of technical replicates per mouse with 95% CI (Two-way ANOVA, Sidak’s multiple comparisons test, n = 5–8).

To further validate the function of TLR7/c-Rel signalling using physiologically relevant primary cells, we generated bone marrow derived dendritic cells (BMDCs) from WT and c-Rel KO C57BL/6J mice. Similar to our *in vitro* system, we found that WT and c-Rel KO BMDCs had similar levels of TLR7 (Fig. 6a). Like c-Rel KO DC2.4s, c-Rel KO BMDCs also showed compromised IL-1β and IL-6 mRNA expression following IMQ stimulation (Fig. 6b). To confirm that the decrease in IL-1β and IL-6 mRNA was also reflected in their protein expression, we confirmed that protein expression of both IL-1β and IL-6 were decreased in c-Rel KO BMDCs following IMQ stimulation (Fig. 6c). Chemical inhibition of c-Rel using PTX in WT BMDCs also inhibited the production of these cytokines (Fig. 6d). As expected, stimulation with CpG and poly (I:C) to stimulate TLR9 and TLR3 was able to induce similar amounts of IL-1β and IL-6 expression in both WT and c-Rel KO BMDCs (Fig. 6e). Taken together, these data further suggest that c-Rel is required for TLR7-induced inflammation in both immortalised and primary DCs.

### c-Rel deficiency compromises the ability of TLR7-primed dendritic cells to polarise naïve T cells to Th17 cells

**Fig. 7:**
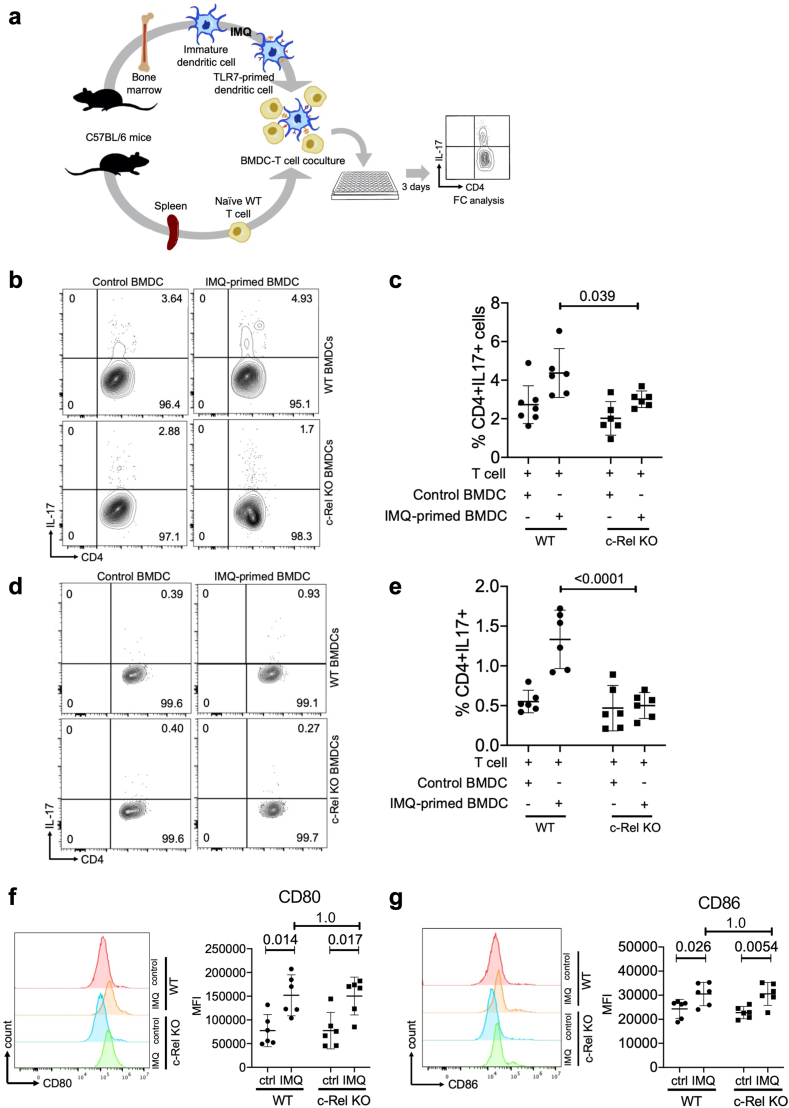
**c-Rel deficient BMDCs have a decreased ability to polarise naïve T cells to Th17 cells.** (a) Schematic of the workflow for IMQ-treated BMDC coculture with naïve WT T cells. (b–e) WT and c-Rel KO BMDCs treated with IMQ for 20 h or control BMDCs were cocultured with naïve WT T cells under (b and c) Th17 polarising and (d and e) Th0 polarising conditions for 3 days. Cells were assessed via flow cytometry for CD4^+^IL17A^+^ population. (c, e) Each data point represents the percentage of CD4^+^IL17A^+^ cells following co-culture under indicated polarising conditions from one mouse (Two-way ANOVA, Sidak’s multiple comparisons test, n = 6–7). (f and g) WT and c-Rel KO BMDCs treated as above were assessed for surface expression of (f) CD80 and (g) CD86 via flow cytometry. Representative histograms (n = 6) are shown (f left, g left). Each data point represents the MFI of the indicated protein from one mouse (f right, g right) (Two-way ANOVA, Sidak’s multiple comparisons test, n = 5–6).

Aberrant crosstalk between DCs and T cells is key in psoriasis pathogenesis. T cell differentiation is determined by cytokines secreted from DCs. IL-6 and IL-1β are among the key cytokines that are required for Th17 differentiation.^72^ Th17 cells are critical in both human and IMQ-induced psoriasis pathogenesis.^24^^,^^73^ Given that c-Rel deficient DCs are unable to produce inflammatory cytokines including IL-1β and IL-6 (Fig. 3, Fig. 6b and c), we hypothesised that the TLR7/c-Rel signalling axis in DCs regulated the induction of naïve T cells to differentiate into Th17 cells during psoriasis. To investigate the functional consequence of the decrease in IL-1β and IL-6 observed in c-Rel deficient BMDCs, we developed an *ex vivo* co-culture system using WT and c-Rel KO BMDCs that were pre-stimulated with IMQ for 20 h and naïve WT T cells (Fig. 7a). We cultured cells for 3 d in Th17-polarising conditions and observed that TLR7-activated WT BMDCs had an increased ability to polarise naïve T cells to Th17 cells compared with non-activated WT BMDC (Fig. 7b, top). We also observed that TLR7-activated WT BMDCs more readily polarised naïve WT T cells to Th17 cells than TLR7-activated c-Rel deficient BMDC (Fig. 7b bottom, 7c). Because *ex vivo* Th17 polarising conditions required the addition of exogenous IL-6 and IL-1β, we repeated our experiments under Th0 polarising conditions to better observe the intrinsic ability of WT and c-Rel deficient BMDCs to induce Th17 differentiation. Similarly, TLR7-activated WT BMDCs had an increased ability to polarise naïve T cells to Th17 cells compared with TLR7-activated c-Rel KO BMDCs under Th0 polarising conditions (Fig. 7d and e).

**Fig. 8:**
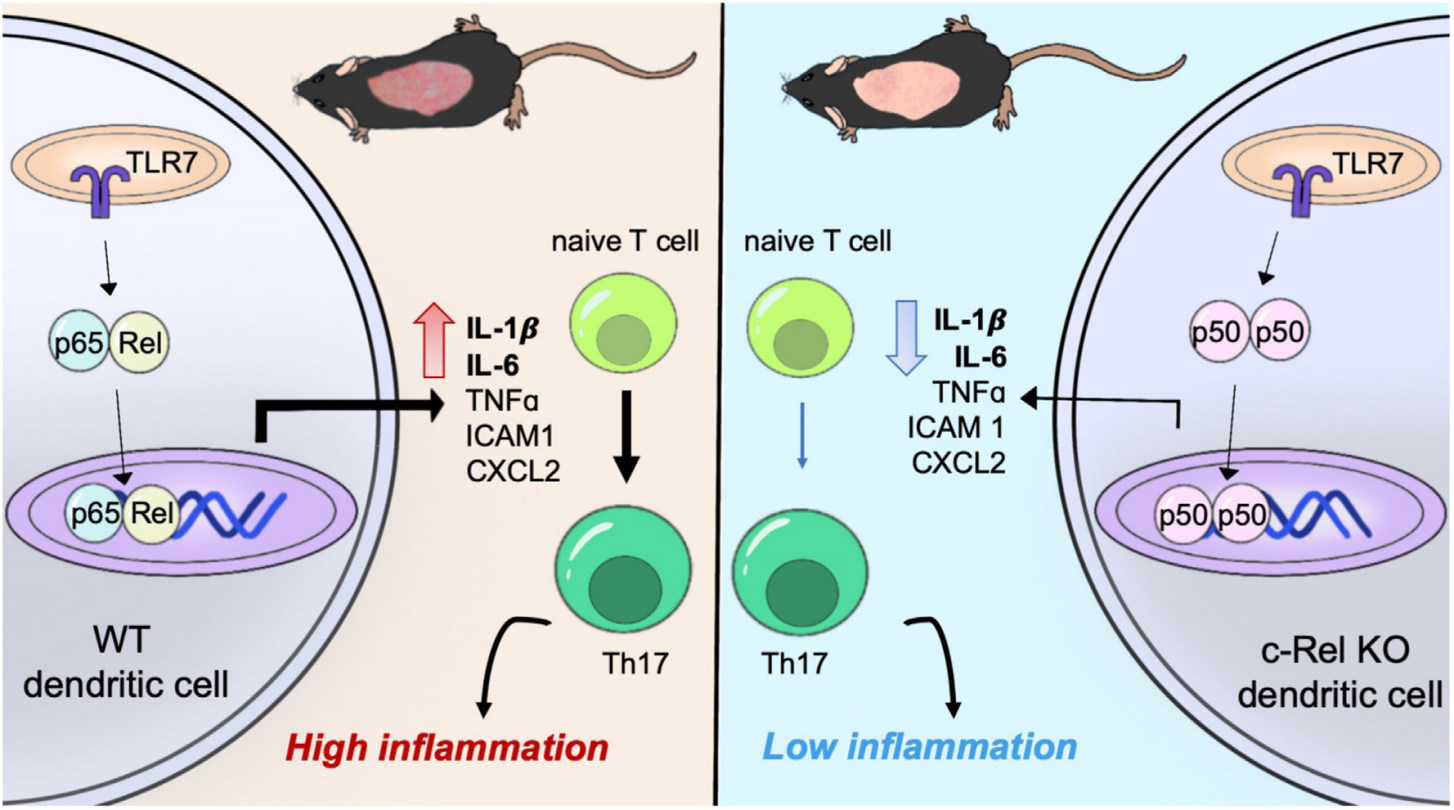
**Schematic summary of the key findings from this study.** (Left) Stimulation of dendritic cells with TLR7 seems to induce p65/c-Rel dimers, which may play a critical role in the transcription of inflammatory genes relevant in psoriasis. (Right) Absence of c-Rel results in enhanced binding of the repressive p50 homodimers to the DNA following TLR7 stimulation, inhibiting the transcription of inflammatory cytokines, thereby protecting the mice from psoriasis.

DCs can influence the differentiation of naïve T cells through secreted cytokines as well as through costimulatory molecules, such as CD80 and CD86, that interact with T cells at the DC:T cell interface.^74^ Therefore, we validated that the decreased ability of c-Rel deficient DCs to induce Th17 differentiation was solely due to hindered DC cytokine expression, and not a difference in the ability of c-Rel KO DCs to activate T cells via expression of costimulatory molecules. We found that both WT and c-Rel-deficient BMDCs had similar basal and IMQ-induced levels of the DC activation markers, CD80 (Fig. 7f) and CD86 (Fig. 7g), suggesting that global DC activation is not affected in c-Rel KO cells. These observations suggest that c-Rel is required for inflammatory cytokine expression in DCs, which in turn regulates the ability of DCs to induce Th17 cell differentiation (Fig. 8, schematic model). Taken together, the unique transcriptional regulatory role of c-Rel controls TLR7-induced DC-mediated inflammatory signalling in the skin.

## Discussion

In this study, we show that NF-κB c-Rel is a transcriptional regulator of TLR7-induced inflammatory signalling in DCs which is critical in psoriasis-like skin inflammation. c-Rel has been reported to be a psoriasis susceptibility locus^75^ and has also been reported to act as both a positive and negative regulator of inflammatory signalling.^31^^,^^45^ Analysis of multiple transcriptomics datasets also showed that c-Rel is upregulated in psoriatic lesions, and c-Rel levels decrease upon treatment with biologics currently used for psoriatic treatment (Fig. 1a–c). *In vivo*, we confirmed the importance of c-Rel in psoriasis development, as our c-Rel deficient mice were protected from IMQ-mediated psoriasiform skin inflammation (Fig. 2). We identified DCs as a key cell type contributing to this phenomenon, and found that chemical inhibition and genetic knockout of c-Rel led to a drastic inhibition of TLR7-induced inflammatory cytokines (Fig. 3). Our mechanistic studies showed how c-Rel dictates NF-κB dimer compositions and binding of specific NF-κB dimers to specific promoters following TLR7 stimulation. TLR7 induces an activating c-Rel/p65 heterodimer in wild-type cells, while c-Rel deficiency increases the p50/p50 inhibitory homodimer binding to the promoters of IL-6 and IL-1β, thus inhibiting their transcription (Fig. 5). Functionally, this impeded TLR7-activated c-Rel deficient DCs in their ability to induce Th17 differentiation (Fig. 7b–e). TLR7 signalling has been implicated in the uncontrolled inflammation associated with psoriatic disease in humans.^44^ In addition, topical IMQ in patients can exacerbate previously controlled psoriasis in both IMQ-treated and uninvolved distant skin sites^27^^,^^28^ further emphasising the role of TLR7 signalling in psoriasis pathogenesis. This study on the role of TLR7/c-Rel signalling axis in DC mediated inflammation during psoriasis provides a comprehensive view of an essential molecular mechanism involved in psoriatic disease pathogenesis.

TLR7 is a receptor for ssRNA and contains two distinct ligand-binding sites: site 1 and site 2. Site 1 recognises nucleoside or base analogues and small molecule agonists including imiquimod while site 2 binds oligoribonucleotides.^58^^,^^76^ In this study, we utilised three chemically distinct TLR7 agonists; IMQ (chemically modified imidazoquinoline small molecule agonist), CL307 (adenine base analogue), and loxoribine (guanine derivative) to stimulate TLR7. All three TLR7 agonists showed a dependence on c-Rel for the production of a subset of proinflammatory genes, suggesting that this observation is not an off-target effect of the compounds used. Moreover, the role of TLR8, a TLR structurally related to TLR7, is debated in mice as some studies have suggested TLR8 to be non-functional in mice^77^ while other studies have found stimulation with a combination of select imidazoquinolines and polyT oligonucleotides can enhance murine TLR8 activation.^78^ Given the differences in receptor specificity between mouse and human TLR8, it is possible that current ligands that agonise human TLR8 are not recognised by the mouse counterpart,^79^ suggesting a current technical limitation in parsing out the differences in the role of c-Rel in TLR7 and TLR8 signalling using murine cells. Our study used only murine DCs for *in vitro* and *ex vivo* studies. Future studies including human DCs, with the utilisation of CRISPR/Cas9 gene editing to delete TLR7/c-Rel axis proteins, will further help to dissect the mechanism and human relevance of this pathway.^80^^,^^81^ In addition, utilising human samples from patients with psoriasis for similar studies will further validate the clinical application of the TLR7/c-Rel signalling pathway as a potential target for therapeutic development for psoriasis. The *REL* SNP rs702873 has been identified as a susceptibility locus in psoriasis,^82^ however, the mechanism in which this SNP is able to alter c-Rel expression or function is unclear. It has been hypothesised that *REL* polymorphisms are able to increase c-Rel protein expression, thereby causing overactive c-Rel-dependent pro-inflammatory signalling.^52^^,^^83^ However, further studies are needed to investigate the clinical outcomes of patients with *REL* SNPs which increase psoriasis susceptibility and c-Rel protein function.

The relevance of the IMQ-induced psoriasis mouse model to human disease has long been discussed.^84^, ^85^, ^86^ The translation of the IMQ-induced psoriasis mouse model to human disease, and thus the necessity of TLR7 signalling in psoriasis, has been questioned due to IMQ treatment inducing psoriasis in TLR7 KO mice.^87^ However, this can potentially be explained by possible TLR7-independent effects of IMQ through its binding to adenosine receptors to trigger inflammation^50^ or to tubulin to inhibit its polymerisation.^88^ Mechanistically, phosphorylation of TLR7 may control its aberrant function, as TLR7 dephosphorylation promotes the exacerbation of psoriatic inflammation in mice.^44^ This suggests a critical role for TLR7 signalling in psoriasis, and warrants future studies on the link between post-translational modifications of TLR7 and c-Rel activation. We acknowledge that as with any disease, it is difficult for a single animal model to capture the many facets of disease pathogenesis. Case reports have shown that clinical application of IMQ on basal cell carcinoma can worsen previously stable psoriasis leading to localised and disseminated psoriasiform lesions^28^ and application of IMQ on nonlesional psoriatic skin has caused the development of psoriasis-like inflammation.^89^ However, as these complications do not arise in all patients using IMQ cream, more research is needed to decipher the molecular mechanisms which trigger aberrant TLR7 signalling and contribute to disease pathogenesis. With this in mind, despite the limitations, our study emphasises the potential role for TLR7 signalling in human psoriasis, and highlights the usefulness of the IMQ-induced psoriasis mouse model as a tool to study the TLR7/c-Rel signalling axis in disease development.

TLR7 activation also upregulates proinflammatory cytokine production^90^ and thus is implicated in fuelling uncontrolled inflammation in human psoriasis.^27^^,^^28^^,^^44^^,^^91^ siRNA targeting of c-Rel has also been shown to ameliorate the severity of IMQ-induced psoriasiform skin modulation,^16^ however this study did not delineate the signalling mechanisms, specific role of TLR7, or the cell types mediating inflammation. Elucidating this current gap in mechanistic insight on the TLR7/c-Rel axis is key in developing better therapeutic strategies for treating psoriasis. Clinically, a wide range of viruses, including HIV, HPV, and HCV have been associated with the development of psoriasis.^92^ However, the mechanisms in which these viruses are associated with psoriasis are unclear. Our study reveals a c-Rel-dependent molecular mechanism regulating DC function following TLR7 agonism that is critical for T cell-mediated hyperinflammation during psoriasis, which may be a potential link for how viral TLR7 activation is involved in worsening psoriatic disease.

Because imiquimod treatment in mice is known to cause systemic inflammation, we hypothesised that our global c-Rel knockout mice that were protected from IMQ-induced psoriatic skin phenotype would also be protected from a systemic inflammatory response. Interestingly, we found that our IMQ treated c-Rel deficient mice also develop systemic inflammation like WT mice, as evidenced by their comparable weight change and splenomegaly (Fig. 2g–h). Thus, it appears that c-Rel function is not critical for generic inflammatory responses elicited by IMQ, which may act through multiple cell types and other NF-κB subunits. To reduce confounding factors, mice were housed in a pathogen-free facility to eliminate possibility random infections and both male and female mice of age between 8 and 12 weeks were used to avoid sex-biased conclusions.

It is possible that the observed psoriasiform skin protection seen in our c-Rel KO mice could be partially caused by cell types other than DCs. For example, the presence of c-Rel deficient keratinocytes or T cells may have contributed to the decreased IMQ-induced psoriasiform phenotype. Our study highlights strong biochemical mechanism of NF-κB pathway signalling following TLR7 activation, however we acknowledge that future studies should utilise mice with a DC-specific deletion of c-Rel to further investigate the cell type specific role of TLR7/c-Rel axis in psoriasis. Epidermal hyperplasia is a key feature of psoriasis and transcription factor signalling cascades in keratinocytes can trigger psoriasis development.^93^ We evaluated the contribution of the TLR7/c-Rel axis to keratinocyte function using the HaCaT cell line and found that both TLR7, as indicated by the response to IMQ stimulation, and c-Rel was not critical for both keratinocyte proliferation and key inflammatory cytokine production. c-Rel has previously been shown to have a role in cell cycle progression, with similar results found in the HaCaT keratinocyte cell line and primary keratinocytes.^46^ This suggests that while c-Rel may play a role in other keratinocyte functions, it likely does not play a role in TLR7-induced cell proliferation. However, we acknowledge that our mechanistic studies were only conducted using the HaCaT cell line and *in vivo* disease mechanisms involve dynamic interactions between primary keratinocytes and multiple other immune and non-immune cell types. Thus, validation of our *in vitro* data using primary cells is extremely important and future studies using keratinocyte-specific c-Rel deficient mice are necessary to fully understand the contribution of the TLR7/c-Rel signalling axis in keratinocytes to psoriasis.

TLR7 stimulation of naïve T cells has been shown to inhibit Th1 and Th17 differentiation.^94^ c-Rel is also required for Th17 cell development in T cells.^15^ To parse out the molecular mechanisms of TLR7/c-Rel signalling specific to DCs, we performed *in vitro* and *ex vivo* assays with immortalised and primary DCs. Our *ex vivo* co-culture system using WT and c-Rel deficient DCs cultured with WT T cells helps tease out the DC specific role of c-Rel in TLR7-induced inflammation. We washed out all TLR7 ligand following initial DC activation to prevent any extraneous effect of IMQ stimulating naïve WT T cells. The decreased ability of TLR7-primed c-Rel deficient DCs to induce Th17 differentiation (Fig. 7b–e) appeared to be specific to the transcriptional role of c-Rel in regulating cytokine production (Fig. 3, Fig. 6b and c) as we found similar levels of DC activation following IMQ stimulation between WT and c-Rel KO DCs (Fig. 7f and g). c-Rel-dependent cytokine production from DCs is critical in Th1 and Th2 polarisation following TLR4 stimulation,^11^ further supporting the potential regulatory role of c-Rel in TLR7-activated DCs for Th17 differentiation. These data suggest that future utilisation of a DC-specific c-Rel KO mouse will be valuable to further delve into potential DC-T cell cross-talk *in vivo* during psoriasis. The interplay between the innate and adaptive immunity is critical to develop and sustain psoriasis.^2^ Consistent with this, we believe that c-Rel is critical in the production of key inflammatory cytokines in DCs needed for Th17 polarisation, thus delineating this specific arm involved in psoriasis pathogenesis.

A vast number of candidate therapeutics have been designed to inhibit pan-NF-κB in order to ameliorate inflammation.^95^ However, as NF-κB is involved in several vital functions, its global inhibition results in more adverse complications than beneficial effects.^96^^,^^97^ Previous studies showed that p65 inhibition *in vivo* ameliorates TLR7-induced skin inflammation.^59^, ^60^, ^61^ Although our biochemical results suggest that TLR7 likely induced an activating c-Rel/p65 heterodimer. p65 inhibition may not be a desirable strategy to adopt in the clinic. Our transcriptomics analysis showed that p65 is not upregulated in human psoriatic lesions (Fig. 1c) and p65 inhibition leads to many unwanted off target effects,^96^ likely due to its ubiquitous role in the regulation of essential cellular functions as evidenced by the embryonic lethality of p65 knockout mice.^98^ In contrast, c-Rel expression and activity is largely associated with cells from haematopoietic lineages^52^ and c-Rel knockout mice show no developmental defects.^9^^,^^10^ c-Rel/p65 heterodimers, as described in this study, are a common activating heterodimer in inflammation.^64^ Thus, c-Rel inhibition may be a viable strategy to selectively hinder NF-κB-mediated inflammation without global inhibition of NF-κB function. As c-Rel inhibitors are well tolerated in mice,^99^ it is more likely that a c-Rel-specific inhibitor may have a better safety profile than a pan-NF-κB or a p65-specific inhibitor.

From a broad perspective, it would be interesting to further explore the role of c-Rel in other biologically relevant diseases involving TLR7, such as systemic lupus erythematosus^100^^,^^101^ and viral infections.^102^^,^^103^ c-Rel deficiency has been shown to be protective against systemic lupus erythematosus (SLE).^104^^,^^105^ Separately, TLR7 agonism has been shown to accelerate SLE^101^ while TLR7 neutralisation protects against lupus nephritis.^106^ Several TLR7 agonists have also been tested as HIV treatment strategies.^107^ Understanding the specifics of the TLR7/c-Rel axis in varying disease contexts is key in obtaining a comprehensive understanding of this relatively less studied pathway, which may aid in developing novel disease-specific therapeutic strategies.

## Contributors

ARL, TJD, TSM, KDC and PR conceived and planned the experiments. ARL, NS, JDC, TJD, AV, JTC, ADG, BR, and PR performed experiments and contributed to the interpretation of the results. ARL and PR wrote the original manuscript. ARL and PR have accessed and verified the underlying data. All authors read and approved the final version of the manuscript.

## Data sharing statement

All the data and methods necessary to reproduce this study are included in the manuscript and supplementary materials. Reagent request will be readily fulfilled following the materials transfer policies of Case Western Reserve University. For inquiries, please contact Parameswaran Ramakrishnan at pxr150@case.edu. A list of publicly-accessible transcriptomic datasets obtained from the GEO database can be found in Supplementary Table S1.

## Declaration of interests

KDC is Vice President-Elect of the American Academy of Dermatology and Treasurer of the International Eczema Council. The authors declare no further potential conflicts of interest.

## Appendix ASupplementary data

Supplementary Figs. S1–S6 and Table S1 Figure S1. **c-Rel expression is increased in lesional skin of patients with psoriasis.** Transcriptomics analysis of c-Rel expression in healthy, nonlesional, and clinical psoriasis samples using public GEO datasets (GSE8710, GSE30999, GSE54456). Each point is representative of mean expression with 95% CI (One-way ANOVA, Tukey’s multiple comparisons test). Figure S2. **Immunohistochemistry staining of c-Rel in negative control skin sections and erythema mean difference calculation**. (a) Representative immunohistochemistry images showing the lack of c-Rel staining of dorsal skin sections of c-Rel KO control and IMQ-treated mice (magnification: 60X, scale bar=50 μm). (b) Graphical representation of the standardised mean difference (SMD) per day from WT and KO erythema and scaling scores. SMD was calculated by the difference in mean outcome between the erythema and scaling scores of IMQ-treated and control-treated mice from each genotype per day, divided by the standard deviation of that genotype per day.^108^ Data are shown as means with 95% CI (multiple unpaired t-tests). Figure S3. **TLR7 stimulation and c-Rel deficiency does not affect keratinocyte function.** (a) WT HaCaT cells were stimulated with IMQ for 0 min, 15 min, and 60 min. Nuclear fractions were prepared and analysed by Western blotting with antibodies against indicated proteins. HDAC2 was used as a loading control. Vinculin was used as nuclear purity control. (b) Densitometry quantification of (a). Protein levels were normalised to HDAC2, then quantified relative to unstimulated (control) lanes. Data are shown as means with 95% CI (n=3-4, Two-tailed unpaired Student’s t-test) (c) Total lysates of WT and c-Rel KO HaCaT cells were probed to assess c-Rel and TLR7 expression. Actin was used as a loading control. (d) WT and c-Rel KO HaCaT cells were stimulated with IMQ for 3 h. Expression of IL-1β and IL-6 was analysed by quantitative real-time PCR using the ΔΔCt method. All values were normalised to the housekeeping gene L32. Data are shown as technical duplicates from at least three separate experiments and represented as means with 95% CI (Two-way ANOVA, Sidak’s multiple comparisons test). (e) WT and c-Rel KO HaCaT cells were stimulated with IMQ for 0 h, 24 h, and 48 h. Cell proliferation was analysed by Cell Counting Kit-8 (CCK8) assay at the timepoints indicated. (Two-way ANOVA, Tukey’s multiple comparisons test, n=8). Figure S4. **c-Rel deficiency does not affect TLR7 expression in dendritic cells.** WT and c-Rel deficient DC2.4s were fixed on coverslips and stained with EEA1 and TLR7. Cells were counterstained with DAPI (magnification: 60X, scale bar=25 μm). Figure S5. **Generation of p65 deficient and p50 deficient DC2.4 cells.** (a) p65 expression in WT and p65 KO DC2.4 cells were analysed by Western blotting. (b) p50 expression in WT and p50 KO DC2.4 cells were analysed by Western blotting. Actin was used as a loading control. Figure S6. **c-Rel deficiency in DC2.4s leads to increased basal levels of other NF-κB subunits.** (a-c). WT and c-Rel KO DC2.4 cells were stimulated with IMQ for 1 h. Nuclear and cytoplasmic fractions were prepared and analysed by western blotting with antibodies against indicated proteins. HDAC2, Sp1 and vinculin were used as loading controls or nuclear and cytoplasmic fraction purity controls. (b) Densitometry quantification of (a). Protein levels were normalised to HDAC2. Data are shown as means with 95% CI (n=3-5, Two-way ANOVA, Sidak’s multiple comparisons test). (d-e) WT DC2.4 cells were transfected with plasmids with either vector control or pCMV-RelB. (d) Whole cell lysates were assessed via Western blotting for RelB expression. Vinculin was used as a loading control. (e) 48 hours following transfection, cells were stimulated with IMQ for 3 h. Expression of IL-1β and IL-6 was analysed by qRT-PCR using the ΔΔCt method. All values were normalised to the housekeeping gene L32. Data are shown as technical duplicates from at least three separate experiments and represented as means with 95% CI (Two-way ANOVA, Sidak’s multiple comparisons test). Table S1. Table representation of public GEO datasets analysed.

EBioMed reagents list final

Full WB all
