# Supplementary material for: NF-κB c-Rel is a critical regulator of TLR7-induced inflammation in psoriasis
Source: eBioMedicine. 2024 Nov 24;110:105452. doi: 10.1016/j.ebiom.2024.105452 (PMC11625363; doi:10.1016/j.ebiom.2024.105452)
Supplement: Supplementary Figs. S1–S6 and Table S1 — Figure S1. c-Rel expression is increased in lesional skin of patients with psoriasis. Transcriptomics analysis of c-Rel expression in healthy, nonlesional, and clinical psoriasis samples using public GEO datasets (GSE8710, GSE30999, GSE54456). Each point is representative of mean expression with 95% CI (One-way ANOVA, Tukey’s multiple comparisons test). Figure S2. Immunohistochemistry staining of c-Rel in negative control skin sections and erythema mean difference calculation. (a) Representative immunohistochemistry images showing the lack of c-Rel staining of dorsal skin sections of c-Rel KO control and IMQ-treated mice (magnification: 60X, scale bar=50 μm). (b) Graphical representation of the standardised mean difference (SMD) per day from WT and KO erythema and scaling scores. SMD was calculated by the difference in mean outcome between the erythema and scaling scores of IMQ-treated and control-treated mice from each genotype per day, divided by the standard deviation of that genotype per day.108 Data are shown as means with 95% CI (multiple unpaired t-tests). Figure S3. TLR7 stimulation and c-Rel deficiency does not affect keratinocyte function. (a) WT HaCaT cells were stimulated with IMQ for 0 min, 15 min, and 60 min. Nuclear fractions were prepared and analysed by Western blotting with antibodies against indicated proteins. HDAC2 was used as a loading control. Vinculin was used as nuclear purity control. (b) Densitometry quantification of (a). Protein levels were normalised to HDAC2, then quantified relative to unstimulated (control) lanes. Data are shown as means with 95% CI (n=3-4, Two-tailed unpaired Student’s t-test) (c) Total lysates of WT and c-Rel KO HaCaT cells were probed to assess c-Rel and TLR7 expression. Actin was used as a loading control. (d) WT and c-Rel KO HaCaT cells were stimulated with IMQ for 3 h. Expression of IL-1β and IL-6 was analysed by quantitative real-time PCR using the ΔΔCt method. All values were normalised to the housekeeping ge [file mmc1.pdf]

Figure S1

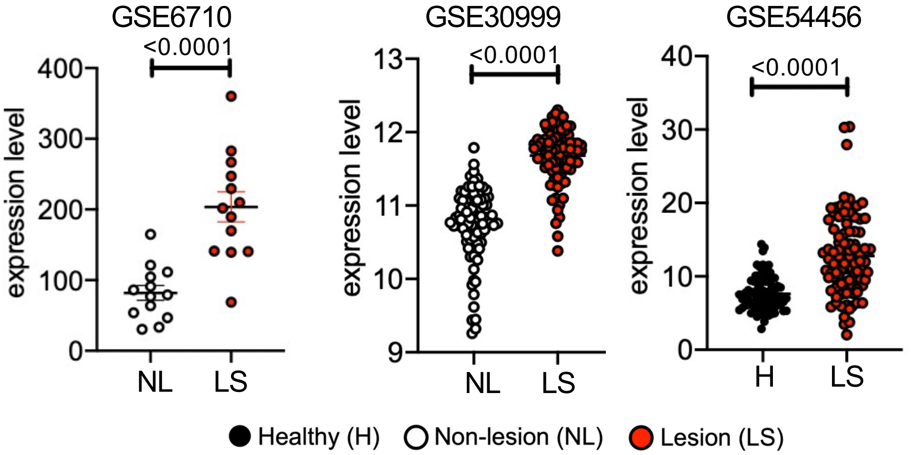

GSE6710
<0.0001
400
300
200
100
0
expression level
NL
LS
GSE30999
<0.0001
13
12
11
10
9
expression level
NL
LS
GSE54456
<0.0001
40
30
20
10
0
expression level
H
LS
Healthy (H)
Non-lesion (NL)
Lesion (LS)

Figure S2

a

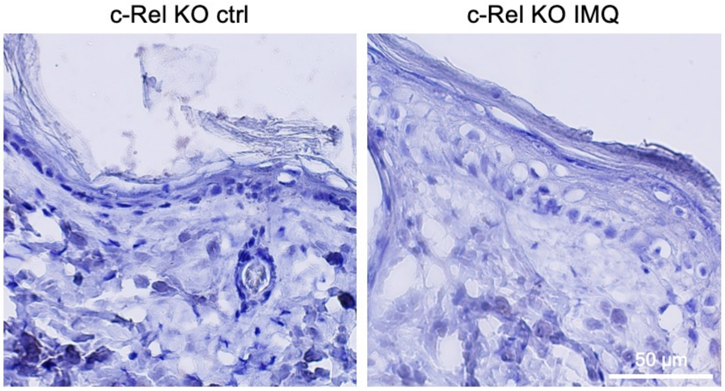

c-Rel KO ctrl
c-Rel KO IMQ
50 μm

b

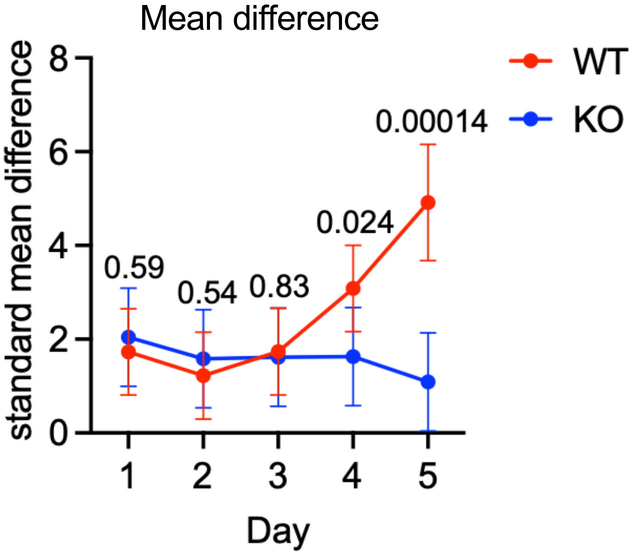

Mean difference
standard mean difference
8
6
4
2
0
1
2
3
4
5
Day
WT
KO
0.59
0.54
0.83
0.024
0.00014

Figure S3

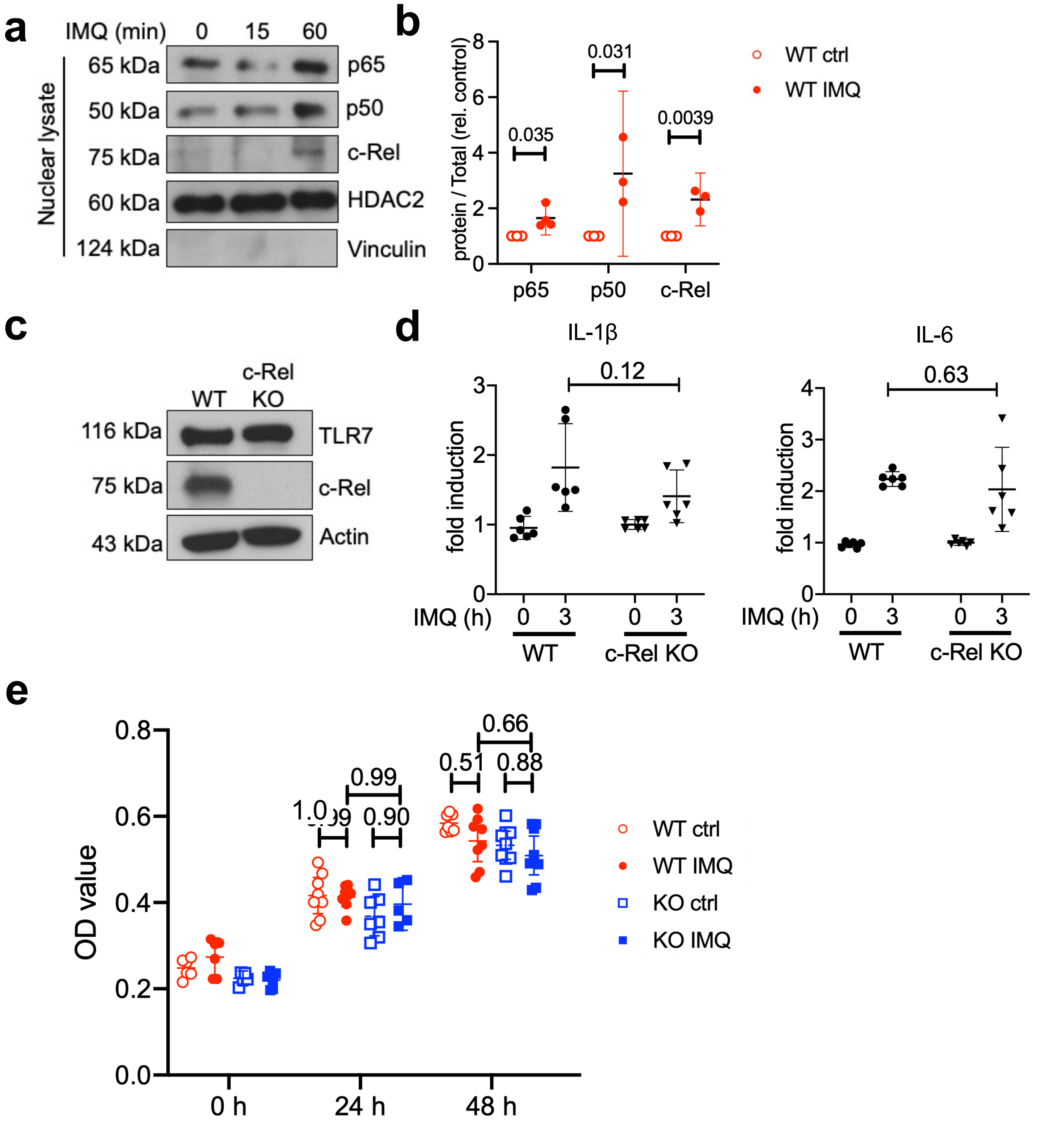

a
IMQ (min) 0 15 60
Nuclear lysate
65 kDa p65
50 kDa p50
75 kDa c-Rel
60 kDa HDAC2
124 kDa Vinculin
b
protein / Total (rel. control)
0.035
0.031
0.0039
p65
p50
c-Rel
WT ctrl
WT IMQ
c
WT
c-Rel KO
116 kDa TLR7
75 kDa c-Rel
43 kDa Actin
d
IL-1β
IL-6
fold induction
0.12
0.63
IMQ (h) 0 3 0 3
WT
c-Rel KO
e
OD value
1.0
0.99
0.90
0.51
0.66
0.88
0 h
24 h
48 h
WT ctrl
WT IMQ
KO ctrl
KO IMQ

Figure S4

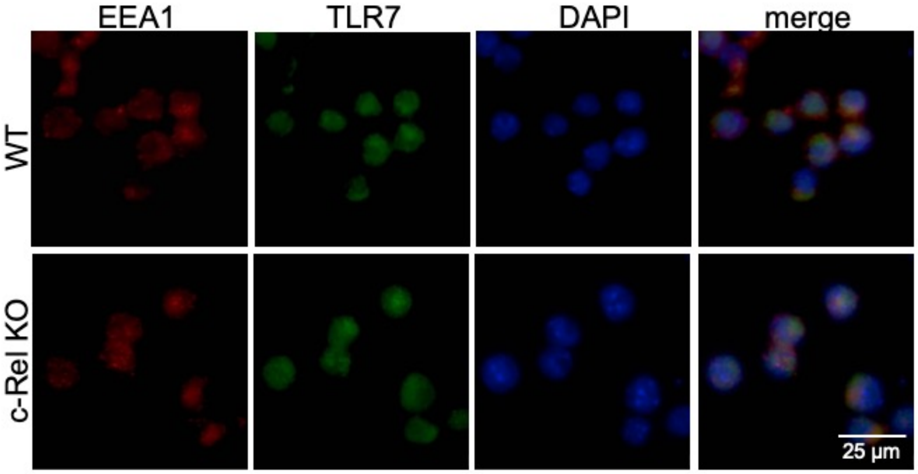

Figure S5

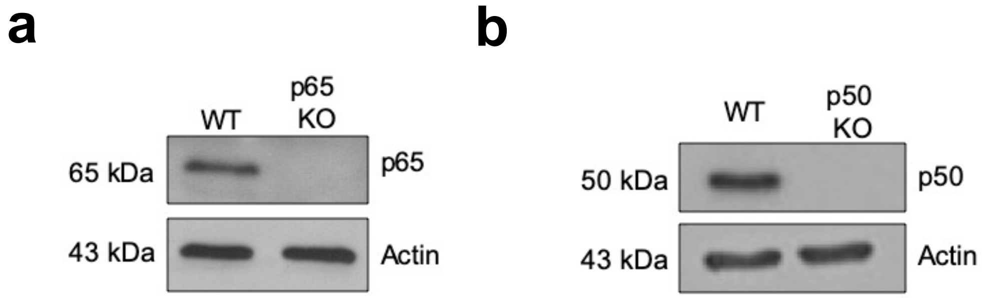
a
WT
p65
KO
65 kDa
p65
43 kDa
Actin
b
WT
p50
KO
50 kDa
p50
43 kDa
Actin

Figure S6

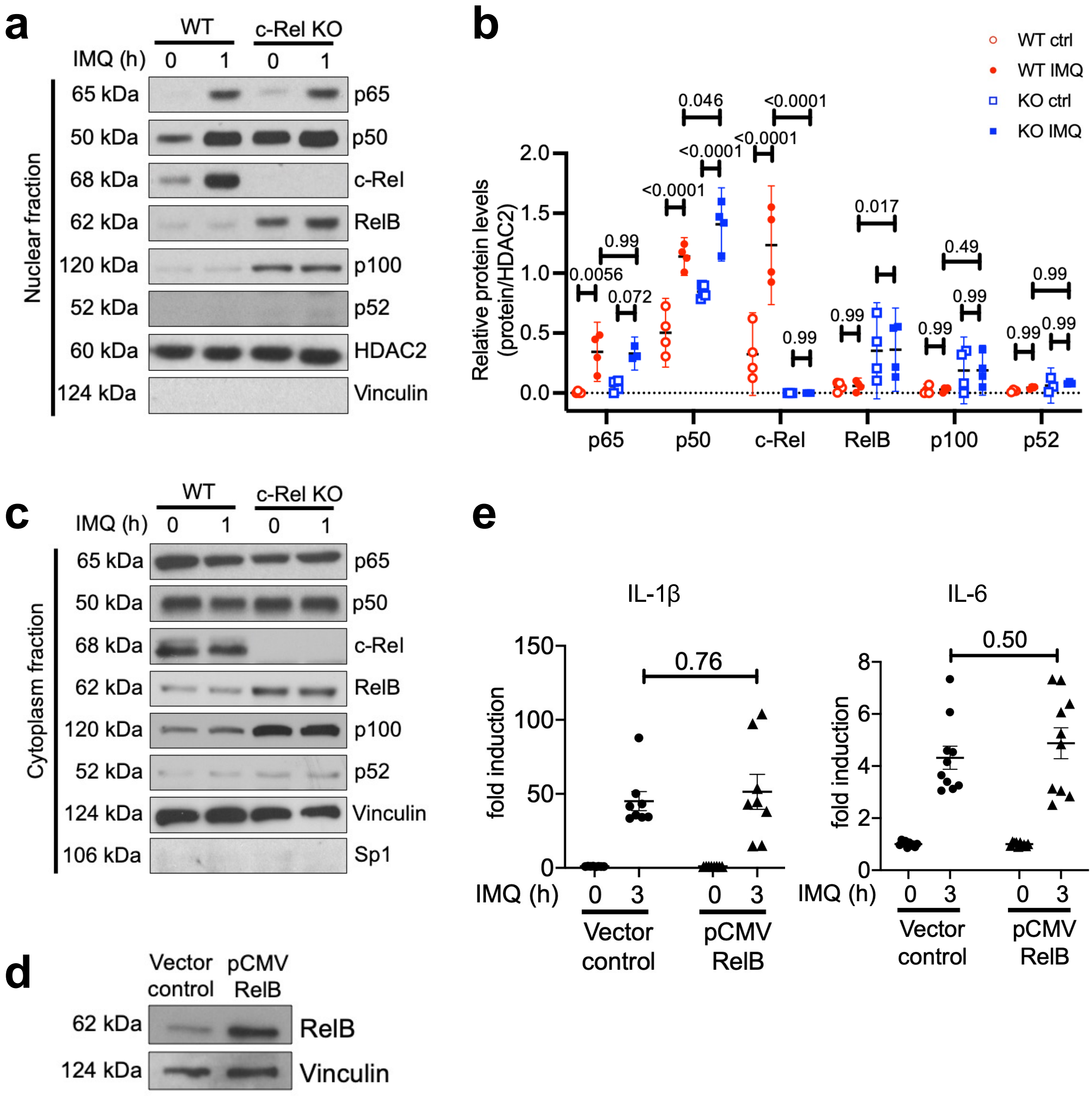

a
WT
c-Rel KO
IMQ (h) 0 1 0 1
Nuclear fraction
65 kDa p65
50 kDa p50
68 kDa c-Rel
62 kDa RelB
120 kDa p100
52 kDa p52
60 kDa HDAC2
124 kDa Vinculin
b
WT ctrl
WT IMQ
KO ctrl
KO IMQ
Relative protein levels (protein/HDAC2)
2.0
1.5
1.0
0.5
0.0
0.0056
0.99
0.072
<0.0001
0.046
<0.0001
<0.0001
<0.0001
0.99
0.99
0.017
0.99
0.49
0.99
0.99
0.99
0.99
p65 p50 c-Rel RelB p100 p52
c
WT
c-Rel KO
IMQ (h) 0 1 0 1
Cytoplasm fraction
65 kDa p65
50 kDa p50
68 kDa c-Rel
62 kDa RelB
120 kDa p100
52 kDa p52
124 kDa Vinculin
106 kDa Sp1
d
Vector control
pCMV RelB
62 kDa RelB
124 kDa Vinculin
e
IL-1β
IL-6
fold induction
150
100
50
0
0.76
8
6
4
2
0
0.50
IMQ (h) 0 3 0 3
Vector control
pCMV RelB

Table S1

GEO accession	Species	Patient groups	Treatment	Type	PMID
GSE13355	human	Healthy<br>Non-lesion<br>Psoriatic lesion	N/A	Transcriptomics	19169254
GSE14905	human	Healthy<br>Non-lesion<br>Psoriatic lesion	N/A	Transcriptomics	18648529
GSE6710	human	Non-lesion<br>Psoriatic lesion	N/A	Transcriptomics	16858420
GSE30999	human	Non-lesion<br>Psoriatic lesion	N/A	Transcriptomics	27667537
GSE54456	human	Healthy<br>Psoriatic lesion	N/A	Transcriptomics	32132203
GSE11903	human	Non-lesion<br>Psoriatic lesion	Etanercept<br>(non-responder and<br>responder)	Transcriptomics	19895991
GSE117468	human	Non-lesion<br>Psoriatic lesion	Placebo<br>Brodalumab<br>Ustekinumab	Transcriptomics	31883845
