# Supplementary material for: NF-κB c-Rel is a critical regulator of TLR7-induced inflammation in psoriasis
Source: eBioMedicine. 2024 Nov 24;110:105452. doi: 10.1016/j.ebiom.2024.105452 (PMC11625363; doi:10.1016/j.ebiom.2024.105452)
Supplement: EBioMed reagents list final [file mmc2.docx]

| **Antibody** | **Source** | **Use** | **Identifier** |
| --- | --- | --- | --- |
| Anti-c-Rel (D4Y6M) | Cell Signaling Technology | WB | Cat#: 12707  RRID: AB_2721030 |
| Anti-c-Rel (D38BS) | Cell Signaling Technology | WB | Cat#: 67489  RRID: AB_2799726 |
| Anti-c-Rel (G7) | Santa Cruz Biotechnology | WB/IP | Cat#: sc-365720  RRID: AB_10846850 |
| Anti-c-Rel (B6) | Santa Cruz Biotechnology | IHC | Cat#: sc-6955  RRID: AB_670194 |
| Anti-p65 (F6) | Santa Cruz Biotechnology | WB/IP | Cat#: sc-8008  RRID: AB_628017 |
| Anti-p65 (C20) | Santa Cruz Biotechnology | WB | Cat#: sc-372  RRID:AB_632037 |
| Anti-p65 (L8F6) | Cell Signaling Technology | IP | Cat#: 6956  RRID:AB_10828935 |
| Anti-p50 (D4P4D) | Cell Signaling Technology | WB/IP | Cat#: 13586  RRID:AB_2665516 |
| Anti- Sp1 (E3) | Santa Cruz Biotechnology | WB | Cat#: sc-17824  RRID:AB_628272 |
| Anti-hnRNPA1 (9H10) | Santa Cruz Biotechnology | WB | Cat#: sc-56700  RRID:AB_629651 |
| Anti-TLR7 (3269) | ProSci | WB | Cat#: 3269  RRID: AB_735503 |
| Anti-TLR7 (PA587550) | Invitrogen | IF | Cat#: PA5-87550  RRID: AB_2804244 |
| PLCγ1 (1249) | Santa Cruz Biotechnology | WB | Cat#: sc-81  RRID: AB_632202 |
| Anti-tubulin (H300) | Santa Cruz Biotechnology | WB | Cat#: sc-5546  RRID: AB_635001 |
| Anti-actin (C4) | Santa Cruz Biotechnology | WB | Cat#: sc-47778  RRID: AB_626632 |
| Anti-lamin A/C (H110) | Santa Cruz Biotechnology | WB | Cat#: sc-20681  RRID: AB_648154 |
| Anti-HDAC2 (680104) | Biolegend | WB | Cat#: 680104  RRID: AB_2632842 |
| Anti-vinculin (938402) | Biolegend | WB | Cat#: 938401  RRID: AB_2876761 |
| Anti-EEA1 (1G11) | Invitrogen | IF | Cat#: 14-9114-82  RRID: AB_2572929 |
| Anti-RelB (C19) | Santa Cruz Biotechnology | WB | Cat#: sc-226  RRID:AB_632341 |
| Anti-p100/p52 (4882) | Cell Signaling Technology | WB | Cat#: 4882  RRID: AB_10695537 |
| Anti-CD11c (N418) | Biolegend | IF | Cat#: 117305  RRID: AB_313774 |
| Anti-CD11c (N418) | Biolegend | FC | Cat#: 301642  RRID: AB_2564083 |
| Anti-IL-6 (MP5-20F3) | Biolegend | FC | Cat#: 504503  RRID: AB_315337 |
| Anti-IL-1β (17-7114-80) | Thermo Fisher Scientific | FC | Cat#: 17-7114-80  RRID: AB_10670739 |
| Anti-CD80 (16-10A1) | Biolegend | FC | Cat#: 104713  RRID: AB_313134 |
| Anti-CD86 (Gl-1) | Biolegend | FC | Cat#: 105005  RRID: AB_313148 |
| Anti-CD4 (GK1.5) | Biolegend | FC | Cat#: 100421  RRID: AB_312706 |
| Anti-IL17A (TC11-18H10.1) | Biolegend | FC | Cat#: 506915  RRID: AB_536017 |

WB: Western Blot; IP: Immunoprecipitation; IF: Immunofluorescence; IHC: Immunohistochemistry; FC: flow cytometry
