# Supplementary material for: NF-κB c-Rel is a critical regulator of TLR7-induced inflammation in psoriasis
Source: eBioMedicine. 2024 Nov 24;110:105452. doi: 10.1016/j.ebiom.2024.105452 (PMC11625363; doi:10.1016/j.ebiom.2024.105452)

Figure 3a

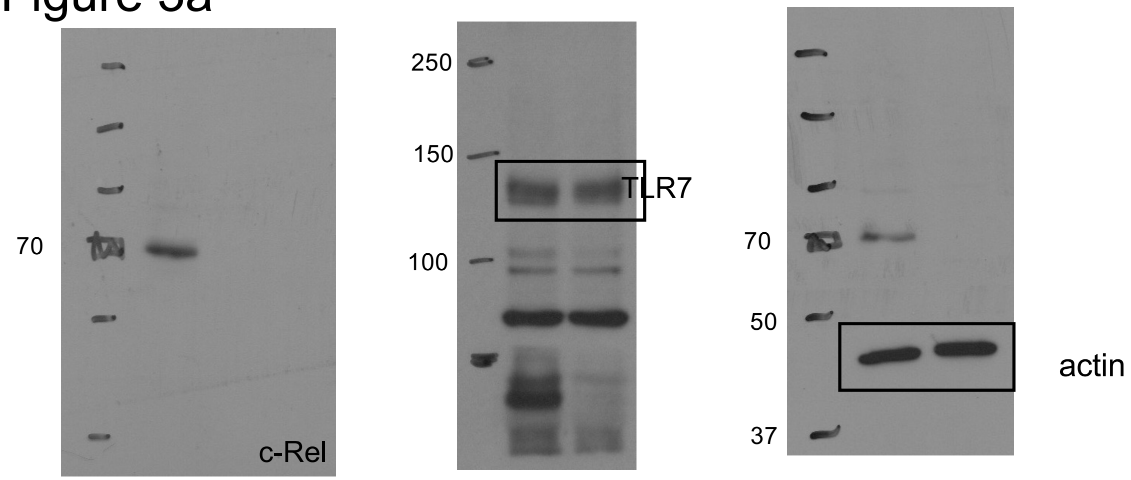

Figure 3f

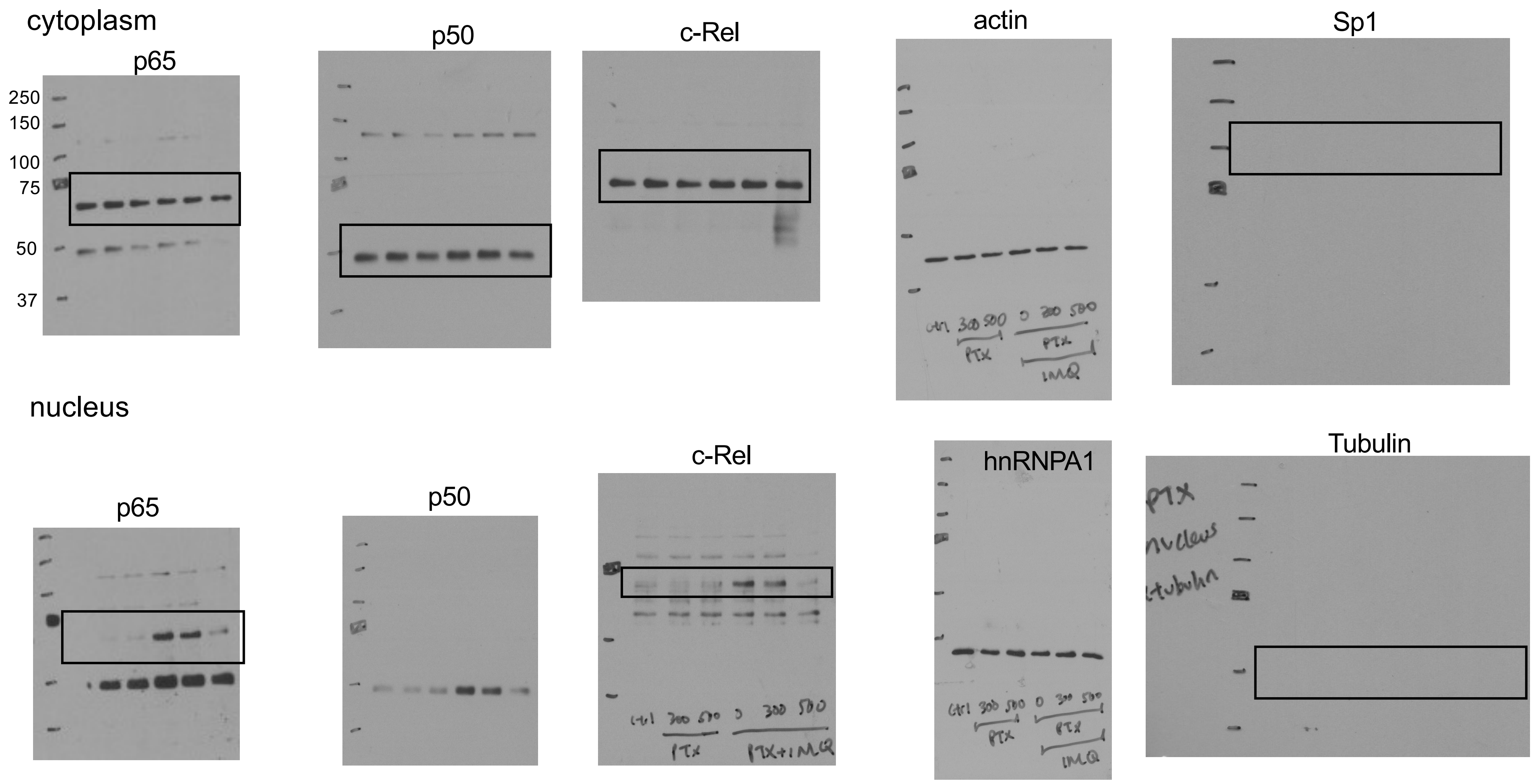
cytoplasm
p65
250
150
100
75
50
37
p50
c-Rel
actin
Sp1
nucleus
p65
p50
c-Rel
hnRNPA1
Tubulin

Figure 5a

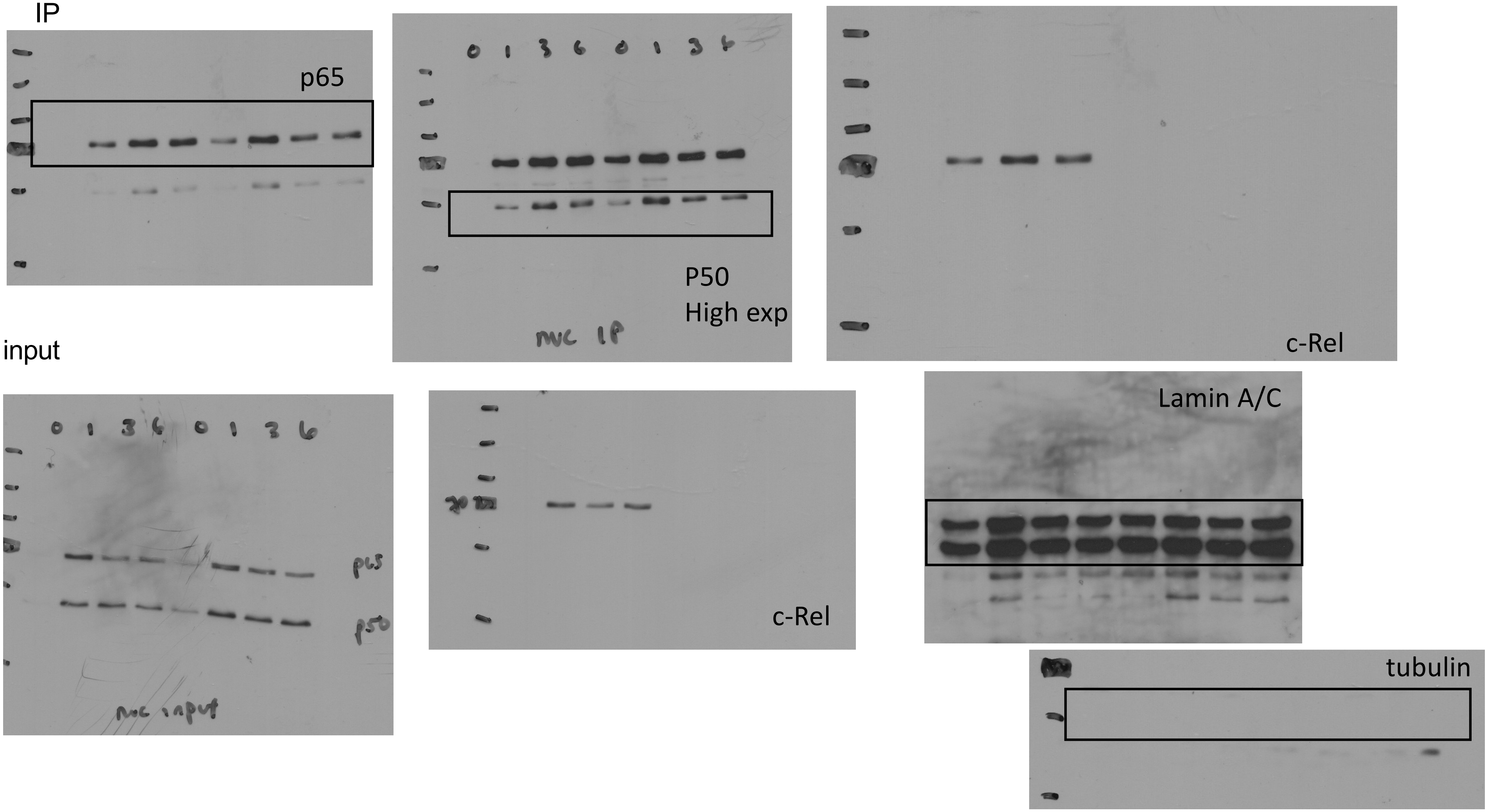

IP
p65
0 1 3 6 0 1 3 6
P50
High exp
c-Rel
input
0 1 3 6 0 1 3 6
p65
p50
c-Rel
Lamin A/C
tubulin

Figure 5b

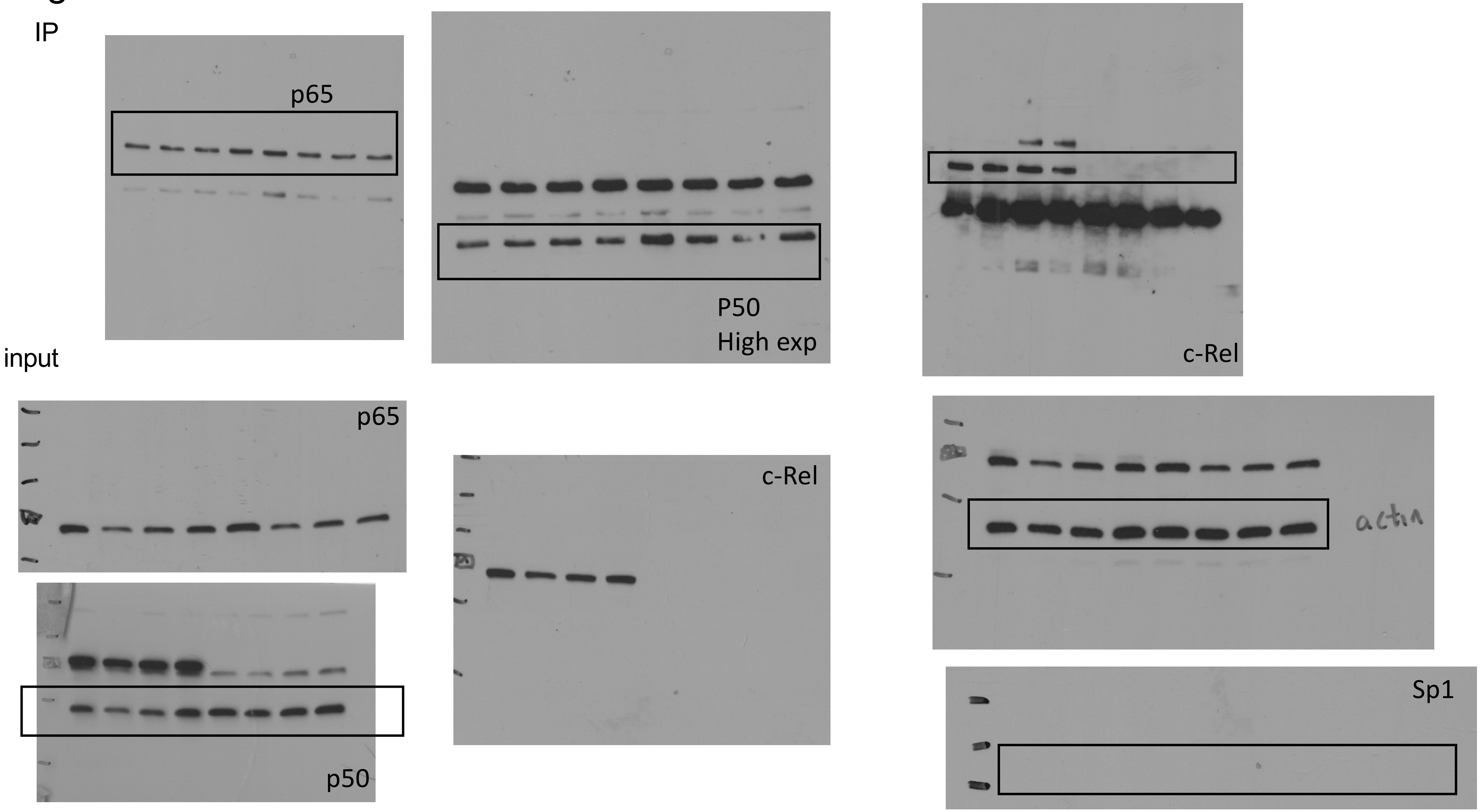

Figure 5c

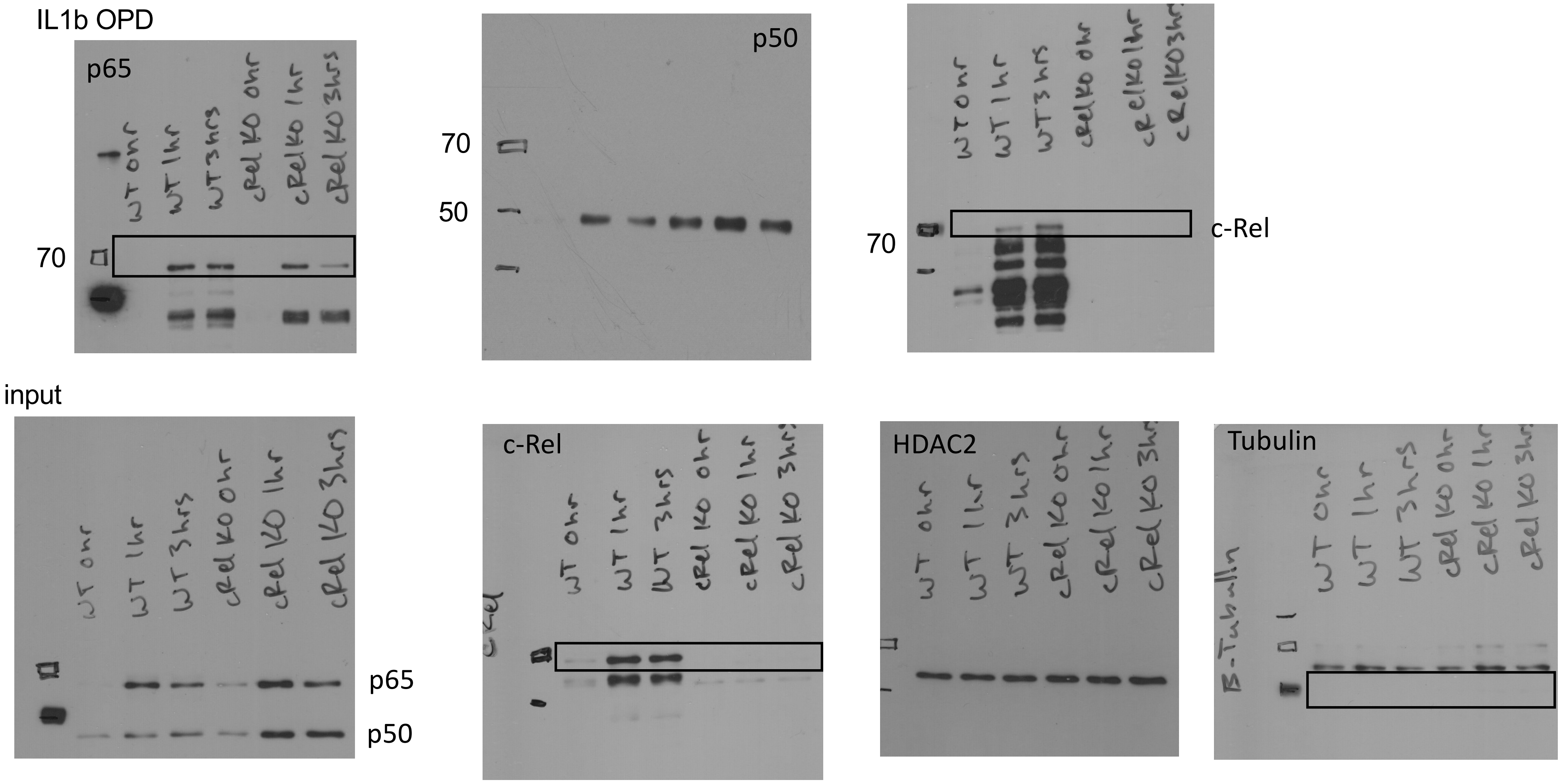
IL1b OPD
p65
70
p50
70
50
70
c-Rel
input
p65
p50
c-Rel
HDAC2
Tubulin

Figure 5d

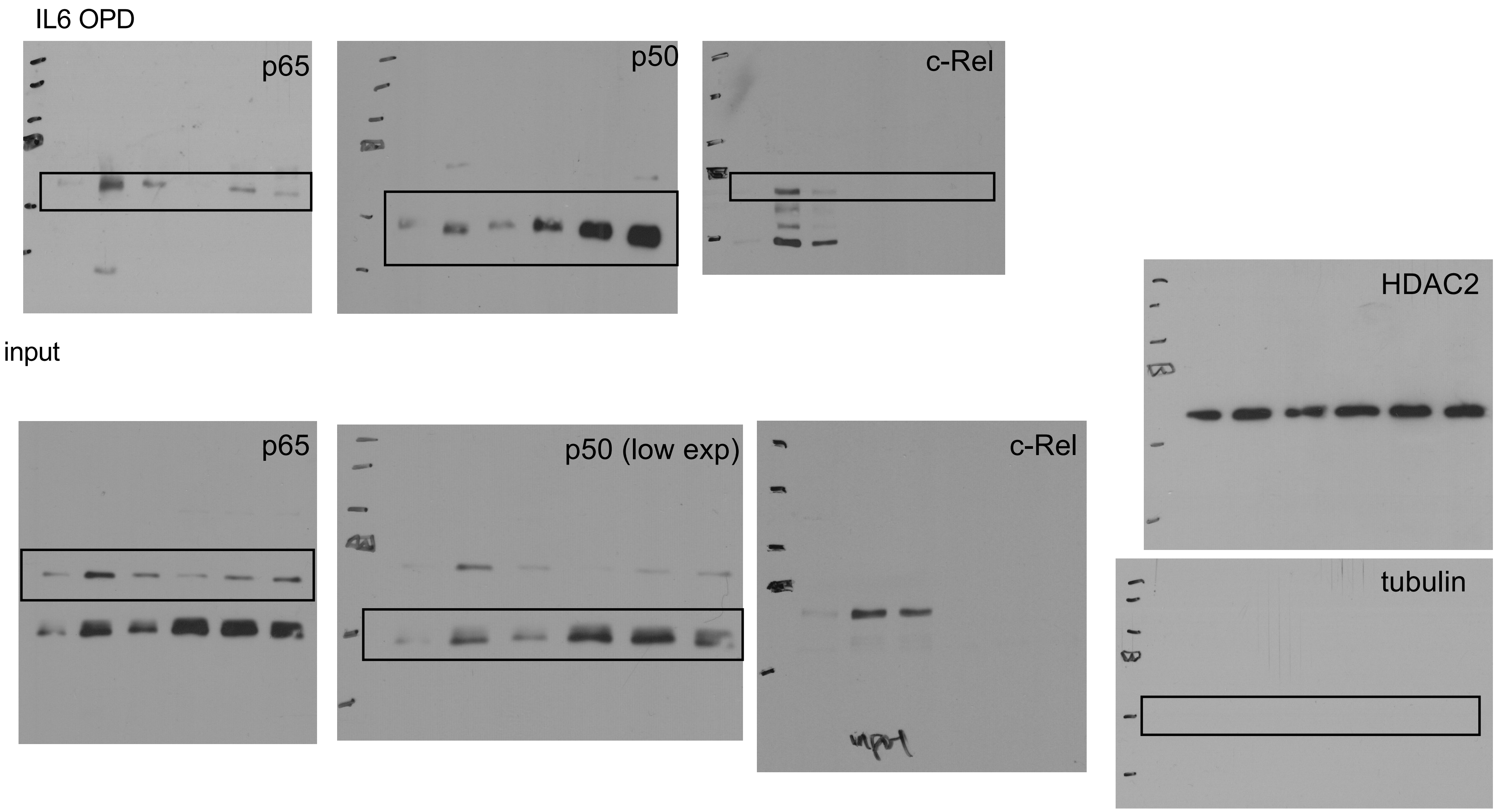

Figure 5e

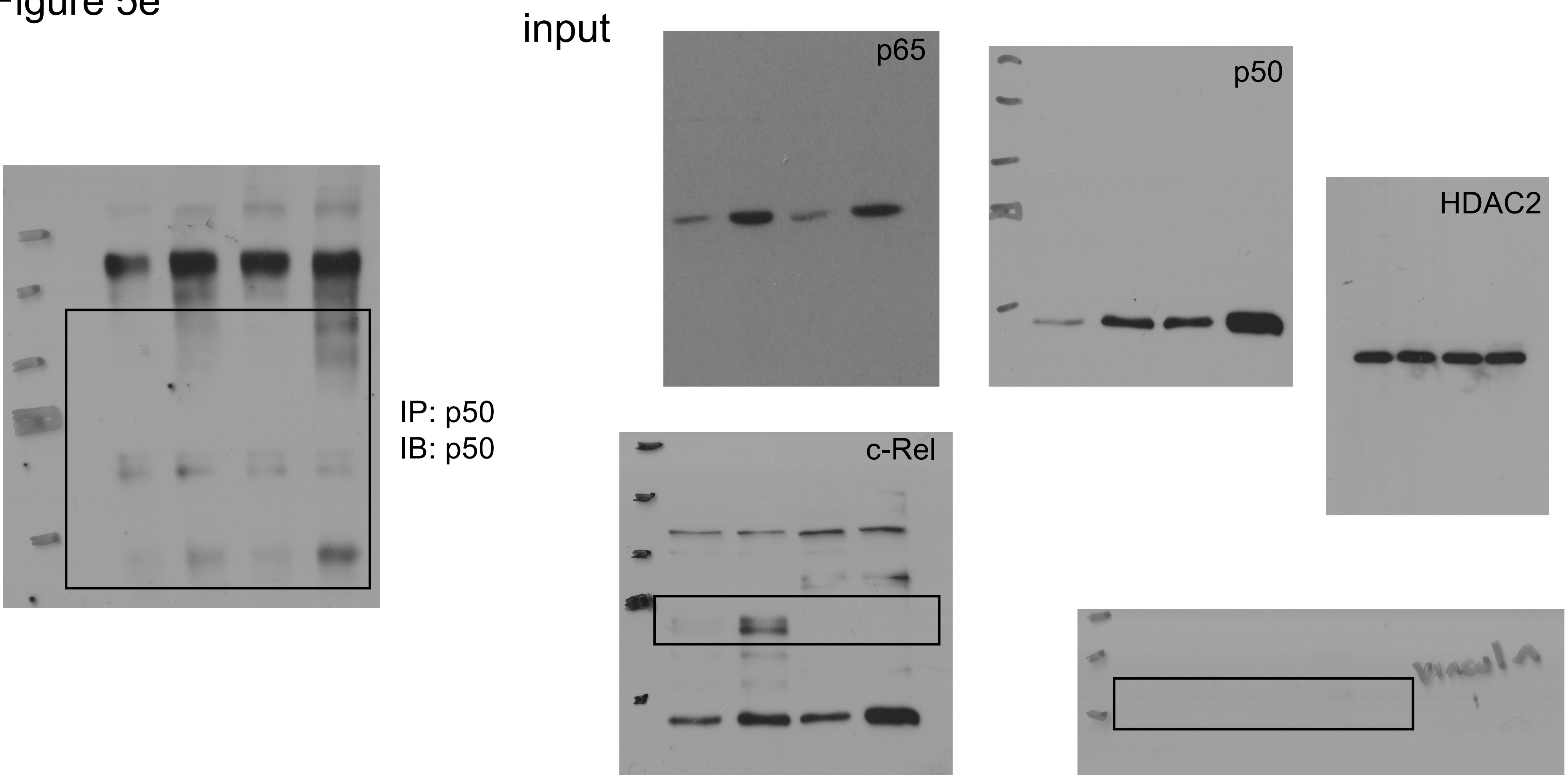

Figure 5g – IP c-Rel

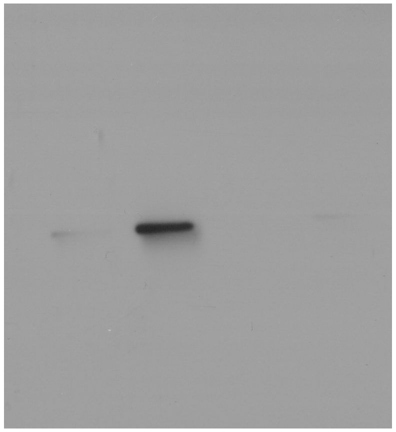

IP: c-Rel
IB: p65

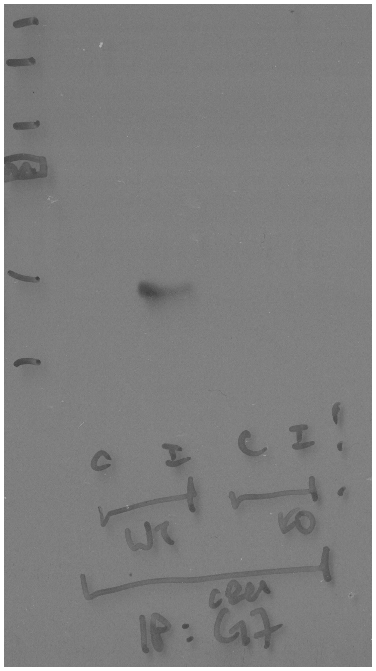

IP: c-Rel
IB: p50

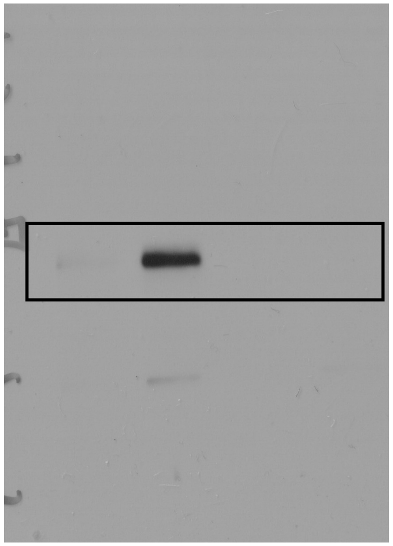

IP: c-Rel
IB: c-Rel

Figure 5g – IP p65

IP: p65
IB: p65

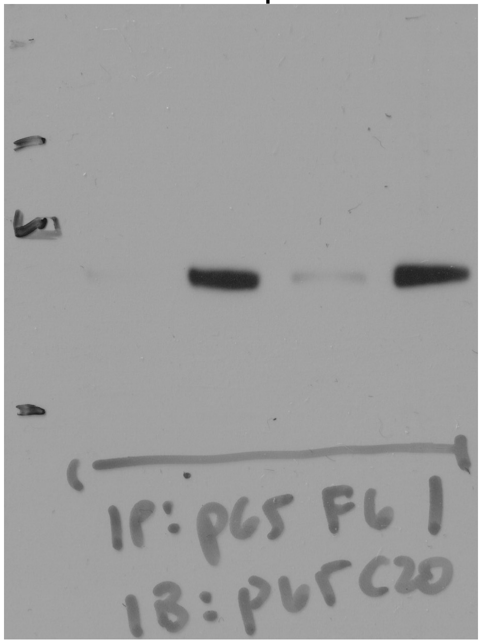

IP: p65
IB: p50

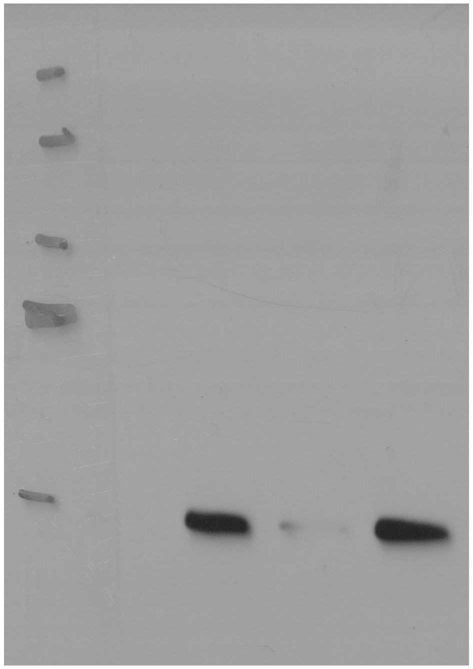

IP: p65
IB: c-Rel

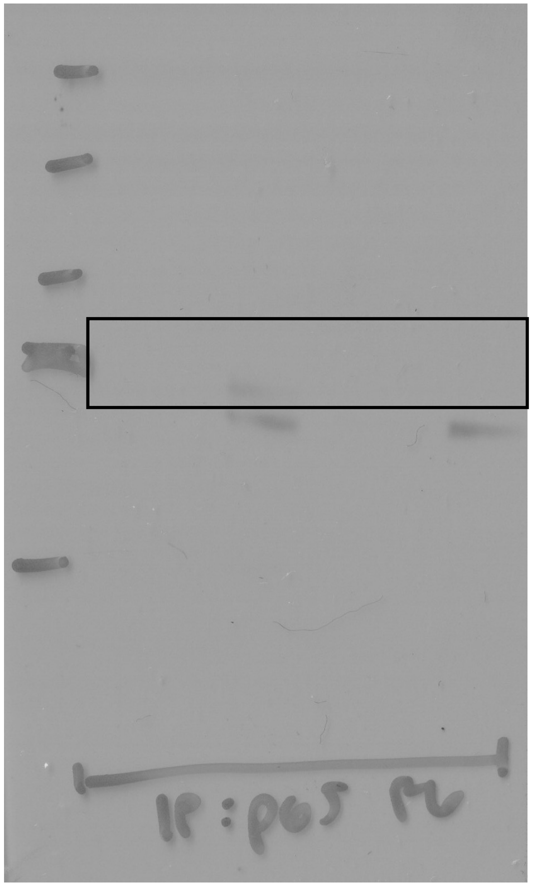

Figure 5g – IL6 OPD following sequential IP

p65

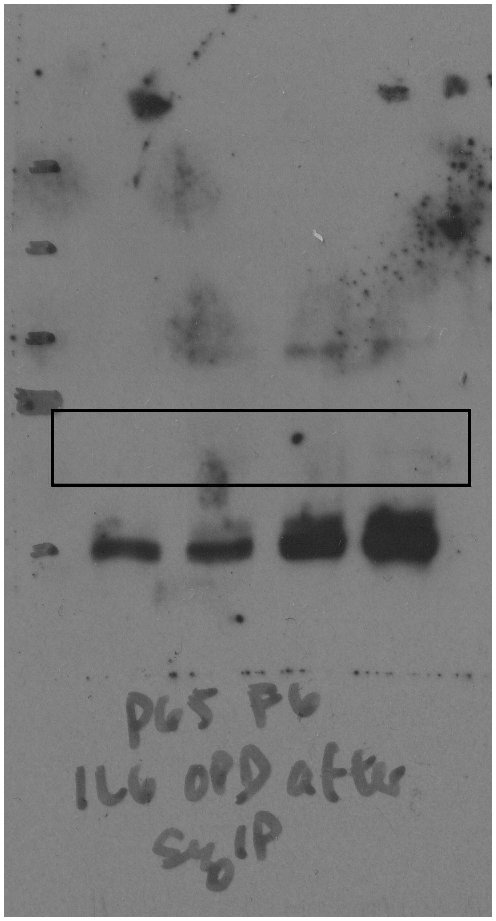

p50

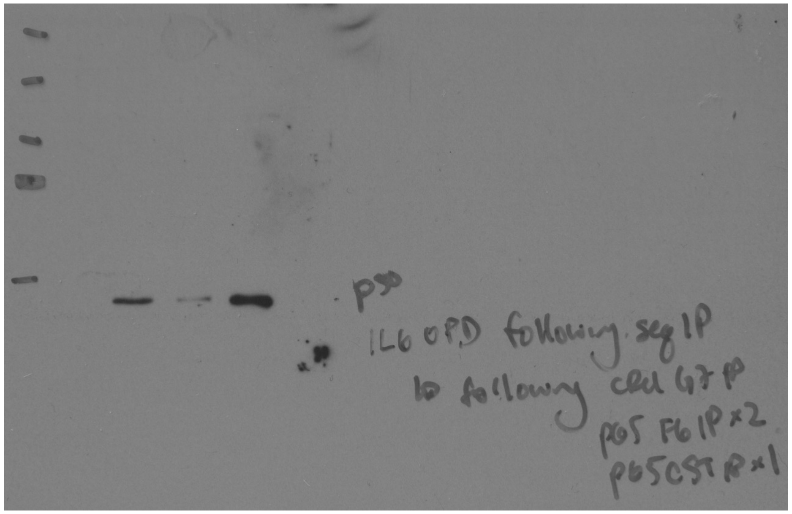

c-Rel

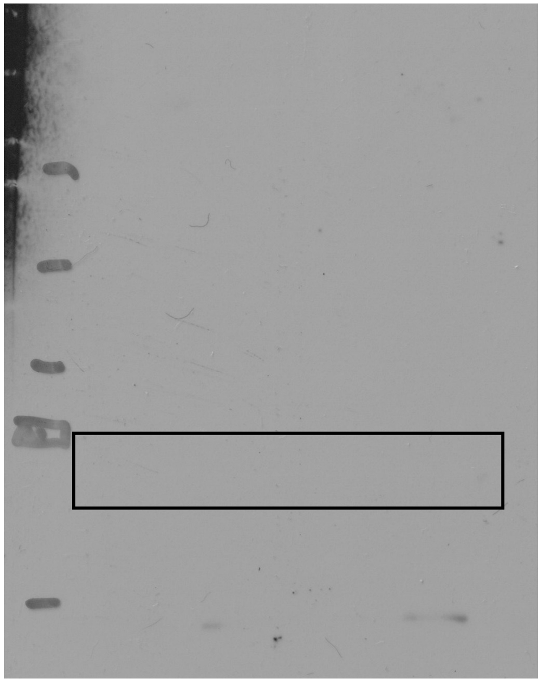

Figure 5g – input

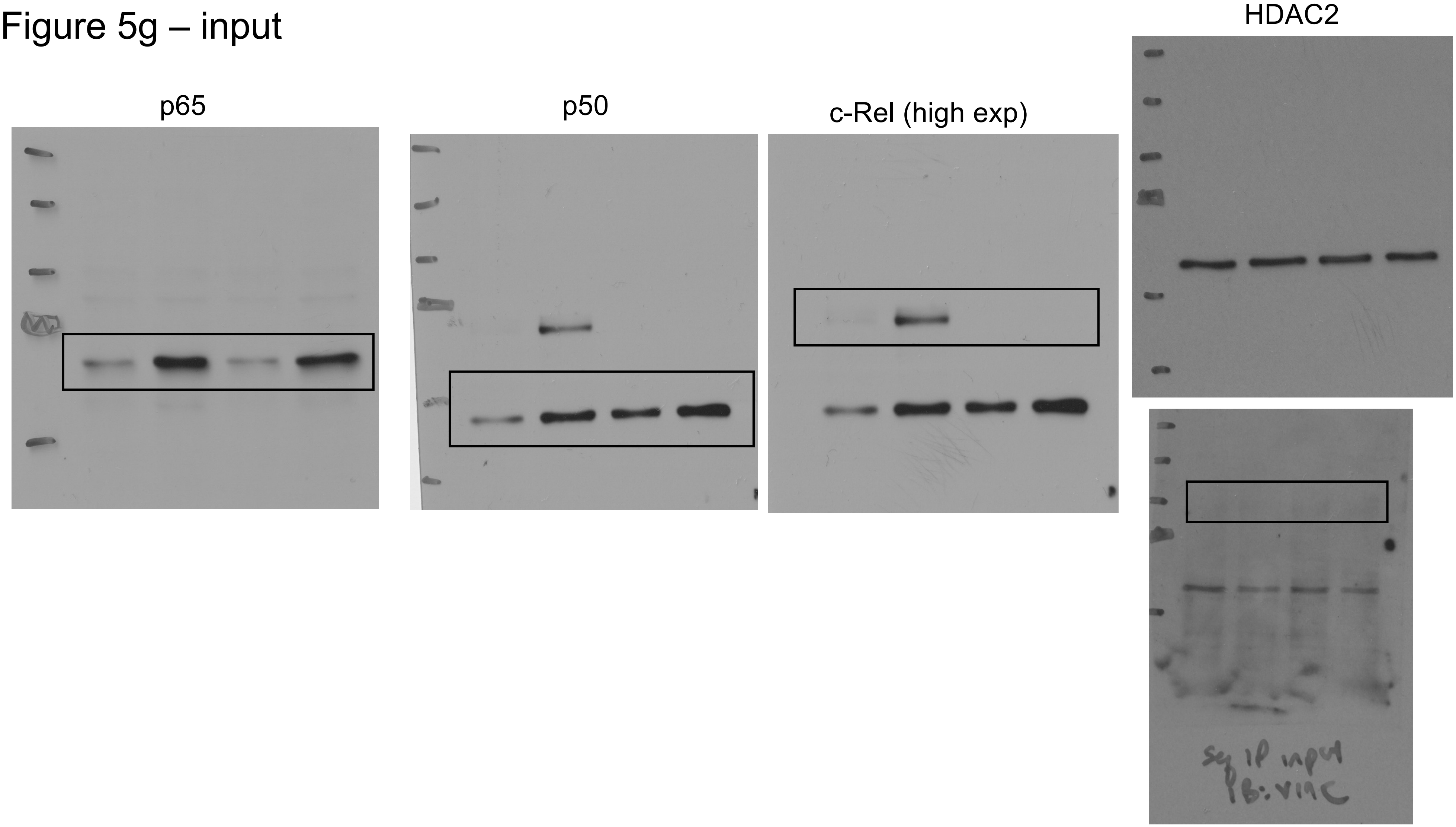

Figure 5h

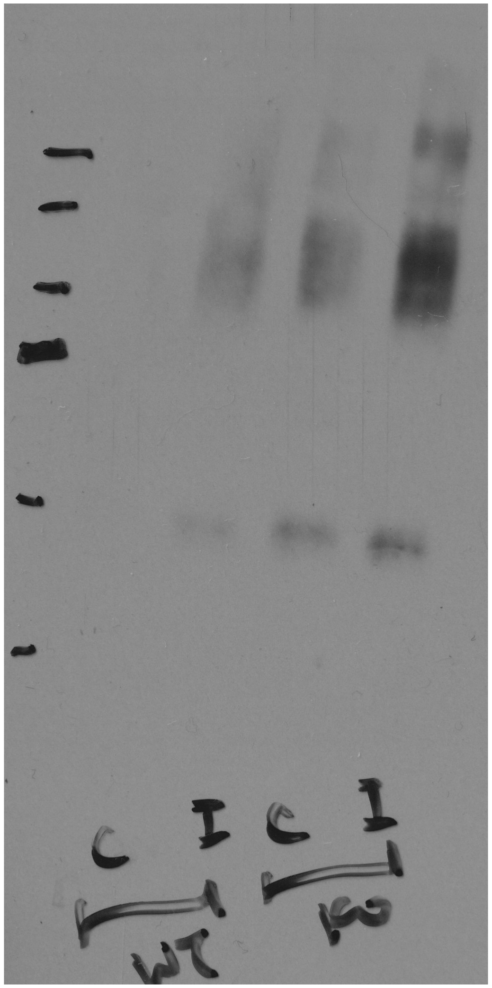

IL6 OPD
Crosslinked
IB: p50

input

p65

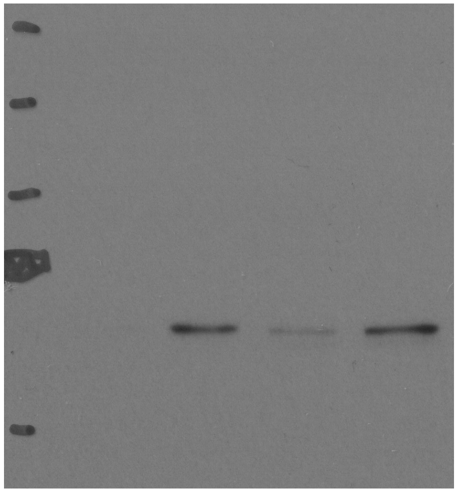

p50

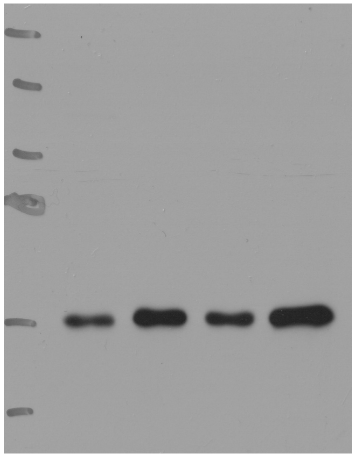

c-Rel

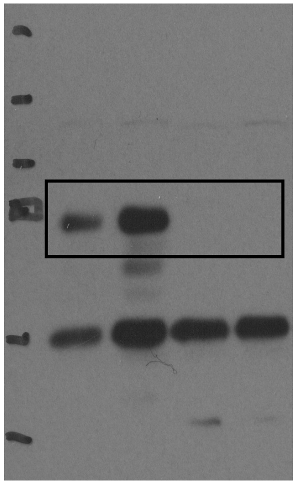

HDAC2

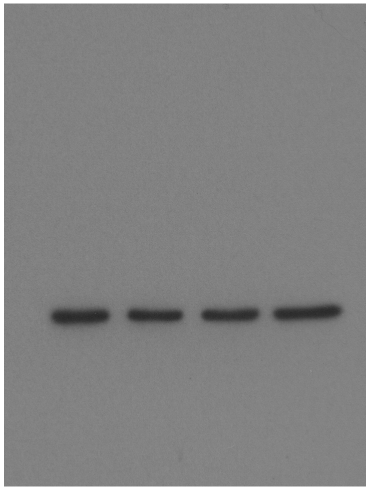

Figure 6

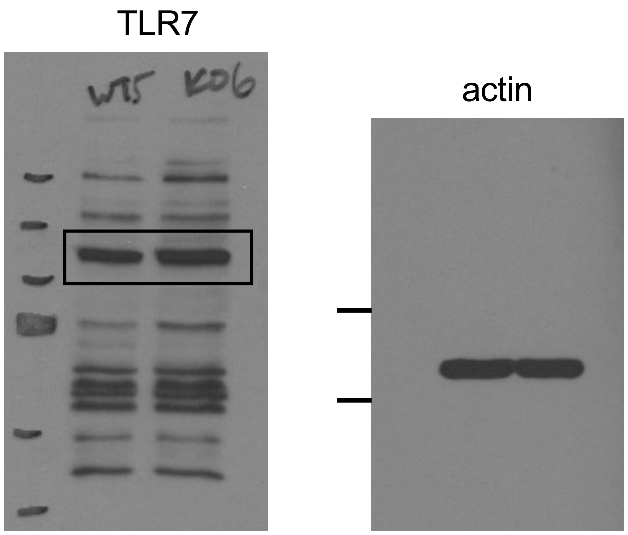

Supplemental Figure 3a

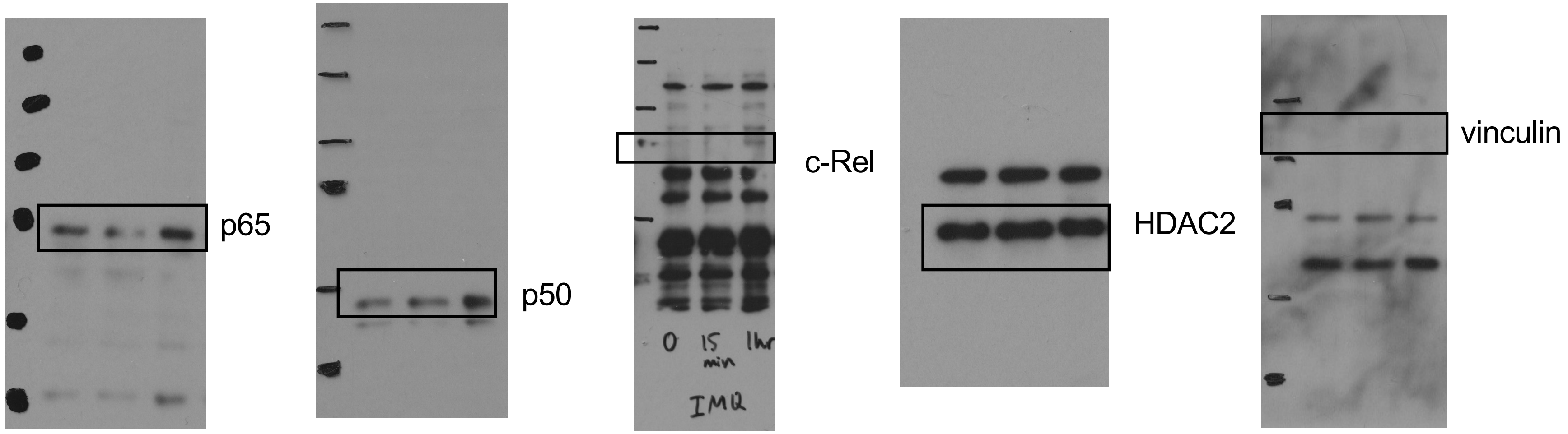

Supplemental Figure 3b

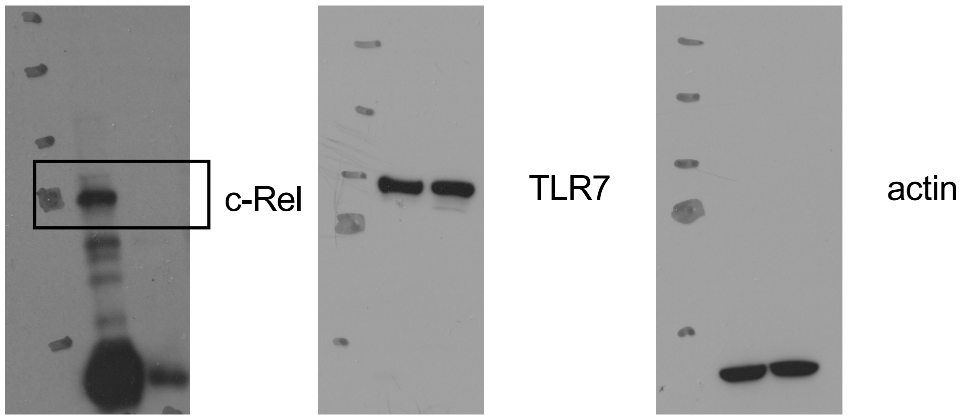

Supplemental figure S5a

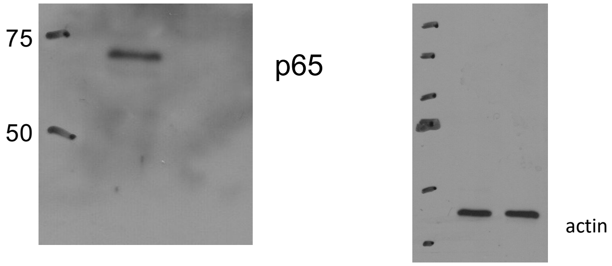

Supplemental figure S5b

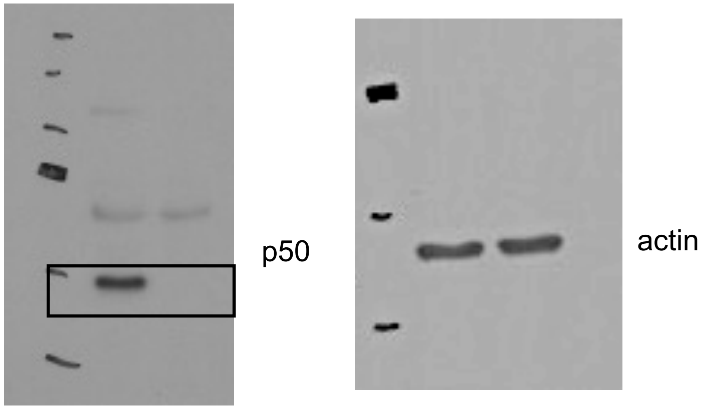

Supplemental Figure 6a

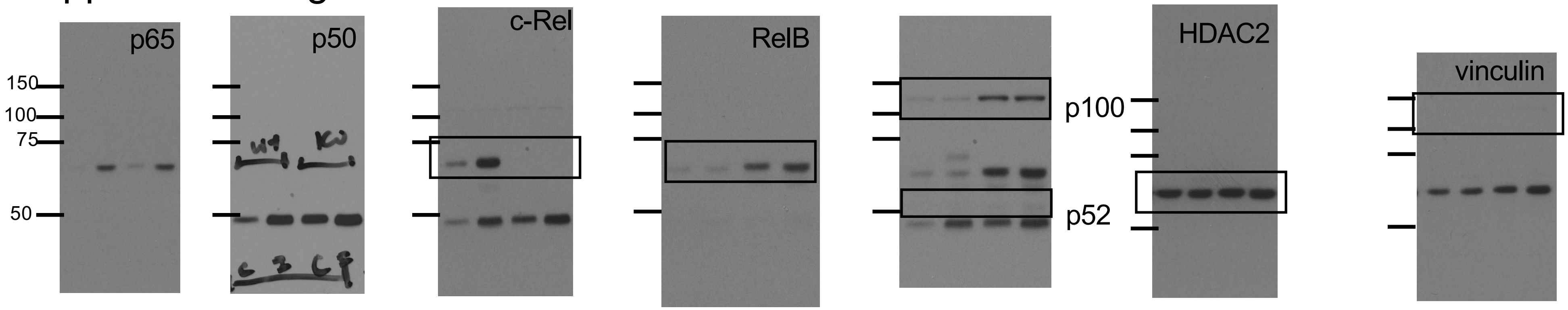

Supplemental Figure 6b

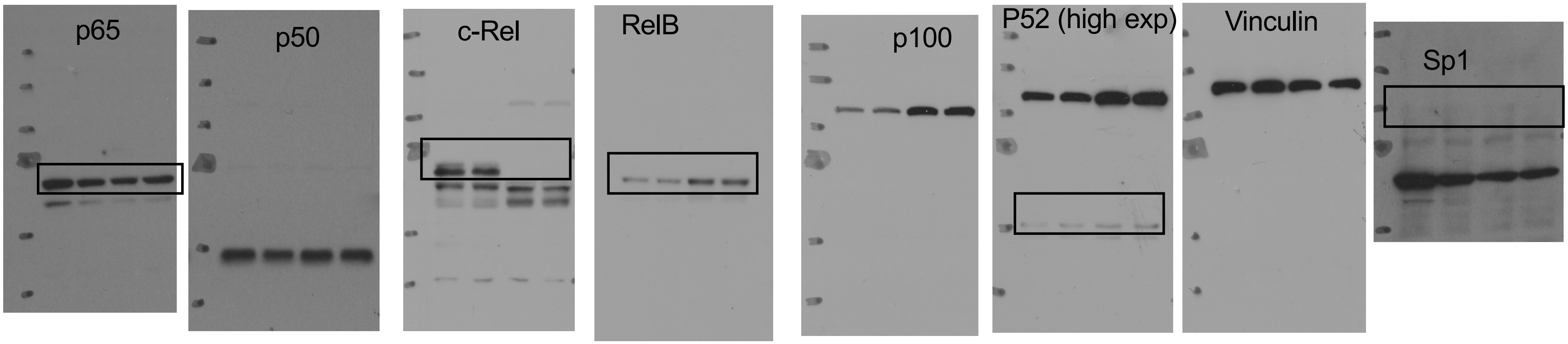

Supplemental Figure 6c

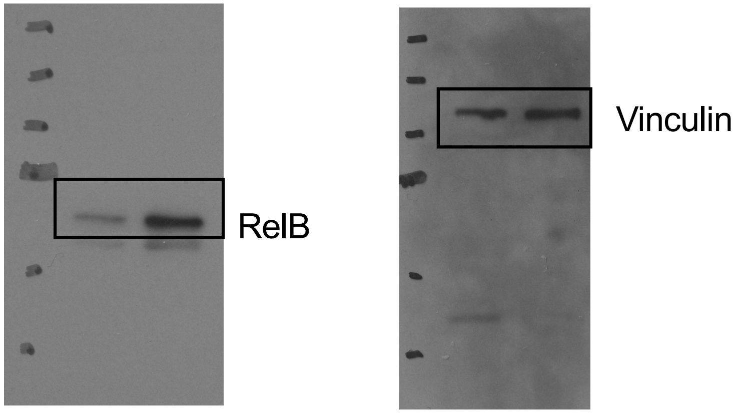

Supplement: Full WB all [file mmc3.pdf]
